# Efficacy and safety of pulsed field ablation and very high-power short-duration ablation for atrial fibrillation: a meta-analysis

**DOI:** 10.3389/fcvm.2026.1860269

**Published:** 2026-07-21

**Authors:** Xiujuan Cao, Jie Hao

**Affiliations:** Department of Cardiology, The Second Hospital of Hebei Medical University, Shijiazhuang, China

**Keywords:** atrial fibrillation, catheter ablation, meta-analysis, pulsed field ablation, very high power short duration ablation

## Abstract

**Background:**

Pulsed field ablation (PFA) and very high power short duration ablation (vHPSD) are new techniques for catheter ablation of atrial fibrillation that have been rapidly developed in recent years. However, there are still limited high-quality studies on the clinical efficacy and safety of the two methods in the treatment of atrial fibrillation.

**Methods:**

We searched PubMed, Embase, Wanfang, CNKI and other Chinese and English databases to collect relevant studies on the efficacy and safety of PFA vs. vHPSD in the treatment of AF published up to May 11, 2026. Two researchers independently completed literature screening and data extraction. We used the Newcastle-Ottawa Scale (NOS) and ROBINS-I tool to evaluate the study quality and risk of bias, respectively. Pooled effect sizes were calculated using random-effects models, and meta-regression and sensitivity analyses were conducted to explore heterogeneity and publication bias.

**Results:**

A total of 11 observational studies involving 1,476 patients were included. Meta-analysis showed that there was no significant difference in the recurrence rate of atrial fibrillation between the PFA group and the vHPSD group during the follow-up period (*RR* = 0.99, 95%CI: 0.91 to 1.07, *P* = 0.722). There was no significant difference in the rate of pulmonary vein isolation between the two groups (*RR* = 1.00, 95%CI: 1.00 to 1.00, *P* = 1.000). In terms of safety outcomes, there was no significant difference in the total incidence of surgery-related complications between the two groups (*RR* = 0.89, 95%CI: 0.58 to 1.35, *P* = 0.5353). In the continuous outcomes, there were significant differences in procedure time (*MD* = −31.64, 95%CI: −40.22 to −23.06, *P* < 0.0001) and fluoroscopy time (*MD* = 6.93, 95%CI: 4.32–9.55, *P* < 0.0001) between the PFA group and the vHPSD group.

**Conclusions:**

Based on the existing research evidence, this study indicates that there is no significant difference in the efficacy and safety of catheter ablation for atrial fibrillation between pulsed field ablation (PFA) and very high power short duration ablation (vHPSD). The procedure time of the PFA group is shorter than that of the vHPSD group, and the fluoroscopy time is longer than that of the vHPSD group. In view of the small sample size and short follow-up time, more randomized controlled trials with large sample size and long-term follow-up are still needed to further verify the above conclusions.

**Systematic Review Registration:**

https://www.crd.york.ac.uk/PROSPERO/view/CRD420261322321, PROSPERO (Registration ID: CRD420261322321).

## 1 Introduction

Atrial fibrillation (AF) is one of the most common arrhythmias in clinical practice, with a prevalence of 2%–4% in adults worldwide and a significant upward trend with age, which has become an increasing public health burden (1). The pathogenesis of AF is extremely complex, which is mainly related to anomalous pulmonary vein triggering, atrial fibrosis/electrical remodeling and other matrix abnormalities, genetics, autonomic nervous system and other factors (2). Catheter ablation is currently the core method for the treatment of atrial fibrillation, and its core goal is to isolate abnormal pulmonary vein electrical signals and restore normal atrial rhythm (3, 4). Although traditional techniques such as radiofrequency ablation and cryoablation have been widely used in clinical practice, they have the limitations of long procedure time, long fluoroscopy time and high incidence of complications (4). In recent years, pulsed field ablation (PFA) and very high power short duration ablation (vHPSD) have rapidly emerged as new catheter ablation technologies. It provides a new option for the treatment of atrial fibrillation.

**Table 1:** Summary of included studies and participant distribution.

Summary of extracted evidence	
Item	Value
Included comparative studies	11
Total participants	1,476
PFA participants	748
vHPSD participants	728

VHPSD is an innovative and improved form of radiofrequency ablation technology. By using ultra-high power and short-term energy delivery mode, it can rapidly form transmural ablation foci, reduce the damage of surrounding tissues caused by heat conduction, and effectively shorten the procedure time and fluoroscopy time (5–7). PFA is an emerging non-thermal ablation technology in recent years. It applies intermittent high-intensity pulsed electric field to myocardial tissue to cause irreversible electroporation of myocardial cell membrane, thereby destroying abnormal pacing and conduction of myocardial cells, and finally achieving the purpose of ablation (8, 9). This technique has the advantages of high tissue selectivity, less damage to normal tissues such as peripheral blood vessels and nerves, short procedure time, and instant release of non-thermal energy, which has been gradually applied to the clinical treatment of atrial fibrillation.

In recent years, PFA and vHPSD, as two innovative techniques in the field of AF ablation, have accumulated abundant clinical evidence. PFA has been regarded as an innovator since its clinical application, with its theoretical ability to avoid collateral damage to adjacent structures (10), It has triggered strong expectations for the comprehensive iteration of existing radiofrequency systems in pulsed electric field ablation. Correspondingly, with the help of ultra-high power and short-time energy output mode, vHPSD also greatly reduces the procedure time and x-ray exposure on the premise of ensuring the efficacy. In addition, vHPSD has the advantage of being embedded in a mature RF operating platform, which should not be underestimated (5). Based on the working principle of non-thermal electroporation and thermal radiofrequency modification, the clinical practice is pushed to an unavoidable choice: should we directly switch to the new strategy of pulsed electric field with the innate advantage of tissue selection, or continue to rely on the deep optimization of radiofrequency modification modality in the interventional treatment of AF?.

Most of the available evidence-based studies have focused on the comparison between PFA and conventional radiofrequency ablation (standard power 20–40 W or conventional HPSD 45–50 W), or single-arm registry studies of each technique. Head-to-head comparisons between PFA and vHPSD remain limited in the published literature. To date, no high-quality randomized controlled trials directly comparing these two ablation modalities have been published, with most available evidence coming from observational studies and retrospective analyses. More importantly, the existing head-to-head data have great differences in patient inclusion criteria, ablation parameters, and postoperative follow-up protocols, leading to obvious differences and even contradictory conclusions on the primary endpoint outcome. For example, some studies have suggested that PFA is superior to vHPSD in terms of procedural safety and long-term sinus rhythm maintenance, while others have shown that vHPSD is superior in terms of procedural efficiency and immediate success.

At the level of clinical practice, this lag of evidence has brought significant confusion: PFA and vHPSD techniques have been rapidly popularized worldwide in recent years, and more than 100 centers in China have carried out these two techniques. However, due to the lack of unified evidence-based basis, the choice of technology in different centers is very random: Some centers blindly advocate the “tissue selectivity” advantage of PFA and use PFA for all patients with atrial fibrillation, ignoring the high cost of consumable materials. However, some centers are familiar with the operation process of vHPSD, and do not give priority to the theoretically safer PFA even in the face of patients with high risk of esophageal injury. This non-standard technical choice not only causes a waste of medical resources, but also makes some patients miss the optimal treatment plan.

Therefore, when clinicians develop individualized ablation strategies for patients with AF, does PFA have the potential to completely replace the new radiofrequency ablation represented by vHPSD, or will the two methods match different application scenarios according to their own characteristics? Even though a small number of studies have attempted to meta-integrate the two, the robustness of the conclusions is still in doubt due to the small number of included studies, insufficient sample size, and failure to fully adjust the heterogeneity among studies, and the clinical controversy cannot be completely resolved. The aim of this meta-analysis is to fill this gap in evidence-based medicine and provide high-quality scientific evidence for the rational choice of AF ablation techniques. We conducted a systematic search for all clinical studies directly comparing PFA and vHPSD in the treatment of atrial fibrillation to date, comprehensively integrated the existing clinical data of clinical trials comparing PFA and vHPSD in the treatment of atrial fibrillation, and quantitatively synthesized the clinical effectiveness and safety of the two techniques with the help of a random effects model. To provide a more comprehensive and robust quantitative evaluation of the effectiveness and safety of the two ablation techniques, so as to assist the rational selection and individualized decision-making of clinical programs in the face of specific patients.

## 2 Materials and methods

This systematic review and meta-analysis was conducted and reported in accordance with the Preferred Reporting Items for Systematic Reviews and Meta-Analyses (PRISMA) guidelines. The study protocol was registered prospectively on PROSPERO (CRD420261322321).

### 2.1 Study eligibility criteria

We included studies that met the following PICO criteria:

#### 2.1.1 Population

Definite diagnosis of paroxysmal atrial fibrillation or persistent atrial fibrillation (2), age, gender, course of disease and other baseline data were not strictly limited; All patients underwent PFA or vHPSD catheter ablation, and the baseline data of the two groups were comparable. Patients with severe valvular heart disease, heart failure (New York Heart function class Ⅳ), severe liver and kidney dysfunction, coagulopathy, malignant tumors and inability to cooperate with follow-up were excluded.

#### 2.1.2 Intervention

Pulsed field ablation (PFA) for PVI.

#### 2.1.3 Comparator

Very high-power short-duration (vHPSD) radiofrequency ablation for PVI, the ablation target was consistent with the intervention group.

#### 2.1.4 Outcomes

The main outcome measures were atrial fibrillation recurrence rate and acute pulmonary vein isolation rate after blank-period.

The secondary outcomes included procedure time, fluoroscopy time, fluoroscopy dose, and complication rate.

#### 2.1.5 Study design

All randomized controlled trials (RCTS) and cohort studies comparing PFA and vHPSD for AF in any language. The inclusion criteria were that the study needed to provide complete original data, or the effect size and related statistical indicators needed for meta-analysis could be extracted from the original data.

Consensus, reviews, case reports, animal experiments, and studies that could not extract valid data were excluded.

### 2.2 Search strategy

PubMed, Embase, Cochrane Library, Web of science, CNKI, Wanfang, and VIP databases were searched for studies on the efficacy and safety of PFA vs. vHPSD in the treatment of AF published up to May 11, 2026.

The English search terms were: atrial fibrillation, pulsed field ablation, PFA, very high power short duration, etc. (Supplementary Table S1). The search was carried out by combining subject headings with free words, while the references of the included articles were manually searched to avoid missing relevant studies.

### 2.3 Study selection and data extraction

Two researchers independently screened the literature according to the inclusion and exclusion criteria. First, the title and abstract of the literature were read to exclude the literature that obviously did not meet the requirements, and then the remaining literature was further screened by reading the full text. Disagreements during the screening process were resolved through discussion between two researchers or with the assistance of a third researcher.

Two researchers independently completed the extraction of data using a pre-designed standardized extraction form. The extraction content included: basic information of the study (first author, publication year, country, sample size, age, intervention measures, follow-up time); Baseline characteristics of patients (sample size, sex ratio, duration of AF, hypertension, diabetes, left ventricular ejection fraction); Surgical indicators and outcome indicators; After the extraction was completed, two researchers cross-checked to ensure the accuracy and completeness of data extraction.

To assess baseline comparability of the included studies, a meta-analysis was performed separately for each baseline variable. For continuous variables, standardized mean differences (*SMDS*) and 95% confidence intervals were calculated. For dichotomous variables, hazard ratios (*RRS*) with 95% confidence intervals were calculated.

### 2.4 Risk of bias assessment

The risk of bias assessment was completed by two researchers independently, and disagreements were resolved by two researchers or the assistance of a third researcher. We used two complementary tools to comprehensively assess the methodological quality and risk of bias of included studies to ensure the reliability of the evidence: Risk of bias of included studies was assessed by using a tool called ROBINS-I, which was recommended by the Cochrane Collaboration. Risk of bias of each study was classified into four levels: low, moderate, severe and very high. Newcastle-Ottawa Scale (NOS) was used to evaluate the methodological quality of the studies. According to the NOS score, the studies were divided into three quality levels: high quality (≥7 points), moderate quality (5–6 points) and low quality (<5 points).

### 2.5 Statistical analysis

Meta-analysis was performed using R software, and the statistical significance level was set at *P* < 0.05.

For studies reporting medians (interquartile range) and medians (Q1,Q3), we used Wan et al. (54) method estimation method to convert them into mean and standard deviation, implemented by R package estmeansd.

Count data (AF recurrence rate, pulmonary vein isolation rate, complication rate) were expressed by risk ratio (*RR*) and 95% confidence interval (*CI*). Measurement data (procedure time, fluoroscopy time) were expressed as mean difference (*MD*) and 95% confidence interval (*CI*).

In this study, stratified subgroup analysis was conducted according to the main type of AF population included in the study, and the studies were divided into two subgroups: “dominated by paroxysmal AF (proportion of paroxysmal AF ≥60%)” and “dominated by persistent AF (proportion of paroxysmal AF < 60%)” to verify the consistency of the results in different AF populations.

Random-effects model combined effect size (REML method) was used by default to fully consider the potential heterogeneity among studies. The I^2^ statistic was used to evaluate the heterogeneity, and subgroup analysis and meta-regression were used to further explore the potential sources of heterogeneity. At the same time, the fixed effect model and the random effect model were used to compare the results, and the robustness of the combined results was comprehensively verified by the sensitivity analysis of changing the effect size, excluding conference abstracts, and excluding individual studies. Funnel plot was used to visually judge the publication bias, and Egger's test, Begg's test and Duval and Tweedie's trim-and-fill method were used for quantitative verification.

## 3 Results

### 3.1 Study selection

**Figure 1:**
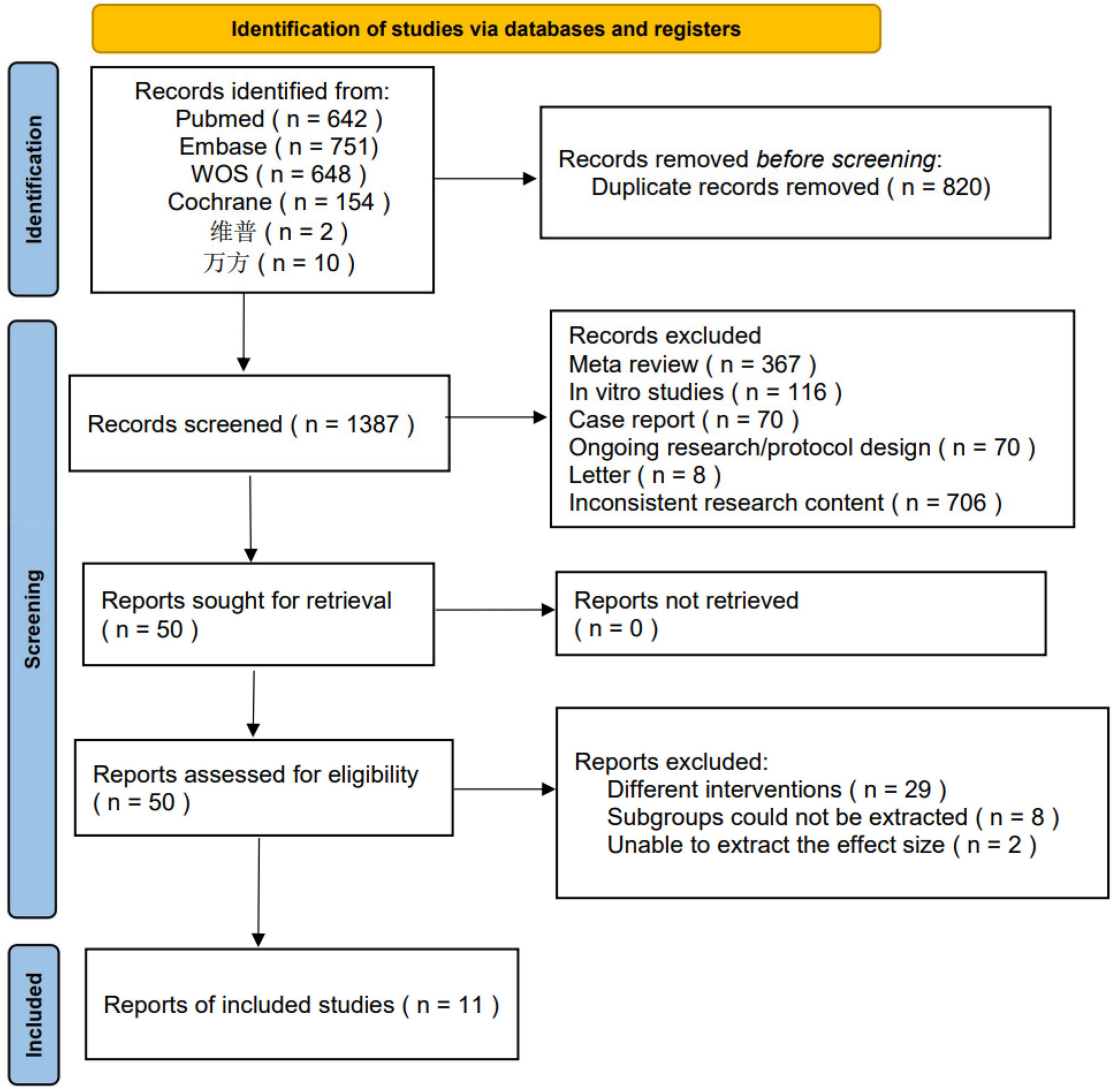
PRISMA flow chart.

A total of 2207 relevant articles were obtained, including 642 from PubMed, 751 from Embase, 154 from Cochrane Library, 648 from Web of Science, 2 from VIP and 10 from Wanfang. EndNote and other literature management tools were used to remove 820 duplicate literatures, and the remaining 1,387 literatures entered the primary screening stage. After reading the title and abstract, we excluded 367 reviews and meta-analyses, 116 *in vitro*/animal experimental studies, 70 case reports, 70 ongoing research or protocol design, 8 reply articles and 706 articles with research content inconsistent with this topic. The remaining 50 articles entered the full-text evaluation stage. After full text reading, 39 studies were excluded for the following reasons: inconsistent interventions (*n* = 29), inability to extract target subgroup data (*n* = 8), and inability to extract effective effect size data (*n* = 2). Finally, 11 studies that met the inclusion and exclusion criteria were included for quantitative combined analysis. The literature screening process is detailed in Figure 1.

### 3.2 Study characteristics

A total of 11 observational studies involving 1,476 patients were included. Based on the above standardized data extraction and cross-checking process, the basic information, baseline characteristics of the patients, and surgery-related indicators of all included studies are summarized in Tables 2–6.

**Table 2:** Study and intervention characteristics.

Study	Country	Publication type	N_PFA	N_vHPSD	PFA technology	vHPSD technology	Follow-u*p* (months)	Monitoring
Campanelli (11)	Italy	Conference abstract	86	119	Farapulse, 2 kV, 8 applications/vein	90 W/4 s posterior wall, 50 W anterior wall	14 (median)	Holter; telephone follow-up
Cardinali (12)	Italy	Conference abstract	24	55	Farapulse, 2 kV, 8 applications/vein; flower configuration on posterior wall	90 W/4 s posterior wall, 50 W anterior wall	14 (9–26)	Holter; clinical follow-up
Dello Russo (13)	Italy	Multicenter retrospective	171	171	Farapulse	QDOT Micro, 90 W/4 s	12 (9–12)	24 h Holter; telephone follow-up
Orban (14)	Hungary	Prospective single-center	87	44	Farapulse	QDOT Micro, 90 W/4 s	6	24 h Holter; clinical follow-up
Popa (15)	Germany	Retrospective single-center	35	32	Farapulse, 2 kV, 8 applications/vein	90 W/4 s	6	ECG; 5-day Holter
Regany-Closa (16)	Spain	Prospective single-center	30	25	Farapulse, 8 applications/vein	90 W/4 s, QDOT	12	ECG; Holter
Santos (17)	Portugal	Prospective single-center	56	45	Farapulse, 8 applications/vein	50 W anterior wall, 90 W/4 s posterior wall, QDOT	12.8 (median 384 days, 341–545)	ECG; Holter; symptom-triggered assessment
Soubh (18)	Germany	Prospective single-center	52	30	Farapulse, 8 applications/vein; 3D mapping	QDOT Micro, 90 W/4 s	6	Holter; PPG; CIED
Weyand (19)	Germany	Observational PSM	100	100	Farapulse	90 W/4 s	3 (90-day blanking period)	48 h Holter; ECG; CIED; remote monitoring; clinical follow-up
Wörmann (20)	Germany	Retrospective single-center	57	57	Farapulse, 8 applications/vein; early stiff guidewire	70 W/7 s; posterior wall 5 s; FlexAbility	4.1 (125 days, 109–162)	NR
Abdullah (21)	United Kingdom	Retrospective single-center	50	50	Farapulse	QMODE+	NR	NR

**Table 3:** Baseline demographic and atrial fibrillation characteristics.

Study	Age, y	Male, %	BMI, kg/m^2^	Paroxysmal AF, %	Persistent AF, %
Campanelli (11)	NR/NR	NR/NR	NR/NR	72/54	22/36
Cardinali (12)	NR/NR	NR/NR	NR/NR	0/0	79.1/78.2
Dello Russo (13)	65 (59–69)/66 (59–71)	63/65	24.7 (24.7–26.2)/24.7 (24.7–26.6)	67/68	33/32
Orban (14)	65 (55–71)/69 (54.8–73)	65.5/68.2	28.5 (25.5–32)/27.7 (25.2–30.7)	59.8/77.3	36.8/22.7
Popa (15)	61.6 ± 11.4/62.2 ± 12.8	68.6/62.5	26.7 ± 4.9/25.4 ± 2.7	100/100	0/0
Regany-Closa (16)	60 (55.05, 64.95)/62.7 (59.6, 65.8)	72/92	NR/NR	87/48	13/52
Santos (17)	61 ± 12/60 ± 11	63/91	28 ± 5/28 ± 4	54/60	46/40
Soubh (18)	67 ± 10/73 ± 7	71/47	28 ± 6/27 ± 6	48/43	52/57
Weyand (19)	65.70 ± 9.46/66.81 ± 9.68	63/54	28.78 ± 5.07/28.31 ± 5.25	46/46	54/54
Wörmann (20)	67 ± 13/67 ± 12	33/40	28 ± 5/27 ± 4	30/30	70/70
Abdullah (21)	63.5 (54.75–69.3)/64.0 (56.0–68.0)	66/70	NR/NR	54/82	46/18

**Table 4:** Comorbidities and echocardiographic characteristics.

Study	Hypertension, %	Diabetes, %	Coronary artery disease, %	LVEF, %	LAVI or LA diameter	CHA₂DS₂-VASc
Campanelli (11)	NR/NR	NR/NR	NR/NR	NR/NR	NR/NR	NR/NR
Cardinali (12)	NR/NR	NR/NR	NR/NR	55.4 (mean)/54.8 (mean)	LAVI 42.6 mL/m^2^/LAVI 42.4 mL/m^2^	3 (0–6)/2 (0–6)
Dello Russo (13)	61/62	23/20	14/13	60 (53–60)/60 (55–60)	LAVI 35 (28–40) mL/m^2^/LAVI 34 (28–28) mL/m^2^	2 (1–3)/2 (1–3)
Orban (14)	73.6/68.2	20.7/9.1	18.4/20.5	55 (51–61)/59 (54–65)	LAVI 54.7 (42.4–65.4) mL/m^2^/LAVI 49 (38.6–57.9) mL/m^2^	NR/NR
Popa (15)	40/50	2.9/9.4	NR/NR	58.5 ± 2.7/57.9 ± 7.8	NR/NR	1.7 ± 1.7/2.0 ± 1.4
Regany-Closa (16)	55/64	3.4/24	NR/NR	59.3 (6.3)/56.1 (7.4)	LA diameter 40.5 (5.6) mm/LA diameter 39.4 (6.1) mm	1.4 (1.1)/1.9 (1.1)
Santos (17)	66/60	21/11	NR/NR	59 ± 10/59 ± 5	LAVI 56 (40–72)/LAVI 51 (38–64)	2 ± 1/1 ± 1
Soubh (18)	83/83	15/13	17/43	52 ± 8/47 ± 14	LAVI 39 ± 14 mL/m^2^/LAVI 48 ± 18 mL/m^2^	4 ± 1.6/4 ± 1.7
Weyand (19)	65/68	14/17	19/16	58.52 ± 5.48/57.78 ± 6.32	LA diameter 46.39 ± 8.15 mm/LA diameter 44.93 ± 8.73 mm	2.34 ± 1.69/2.67 ± 1.62
Wörmann (20)	65/60	16/14	25/19	56 ± 6/56 ± 9	LA diameter 39.6 ± 6.0 mm/LA diameter 38.0 ± 3.5 mm	3 (median)/3 (median)
Abdullah (21)	NR/NR	NR/NR	NR/NR	NR/NR	NR/NR	NR/NR

**Table 5:** Procedural and follow-up outcomes.

Study	Procedure time, min	Fluoroscopy time, min	Fluoroscopy dose	Acute PVI success, %	Total complications, %	Freedom from recu RR ence, %
Campanelli (11)	80 ± 29/108 ± 39	21 ± 8/14 ± 10	NR/NR	100/100	3/6	71/76
Cardinali (12)	92.1 ± 36.9/134.2 ± 36.1	25.5 ± 9.3/17.4 ± 11.5	NR/NR	100/100	4.2/5.5	79.2/78.2
Dello Russo (13)	70 (60–80)/100 (75–120)	15 (13–19)/7 (4–12)	NR/NR	100/100	3/3	75/77
Orban (14)	40 (33–49)/56 (50–67)	8 (5.8–12)/4.9 (2.5–6.6)	180.1 (104.5–282.3) µGy·m^2^/91.1 (45.1–170.9) µGy·m^2^	100/100	3.4/2.3	85.1/81.8
Popa (15)	81.1 ± 26.0/112.8 ± 29.6	18.2 ± 9.5/6.0 ± 2.7	NR/NR	100/100	2.9/3.1	84.4/70
Regany-Closa (16)	NR/NR	NR/NR	NR/NR	100/100	NR/NR	80/84
Santos (17)	88 (66–111)/97 (75–142)	13 (10–16)/5 (3–7)	NR/NR	NR/NR	1.8/0	82/77
Soubh (18)	64 ± 19/99 ± 32	14 ± 6/9 ± 5	14 ± 9 Gy·cm^2^/11 ± 9 Gy·cm^2^	100/100	0/3	91/78
Weyand (19)	43.80 ± 13.13/78.25 ± 25.24	10.34 ± 4.12/7.68 ± 5.04	963.9 ± 833.1 cGy·cm^2^/711.6 ± 617.7 cGy·cm^2^	100/100	1/4	83/97
Wörmann (20)	65 ± 17/95 ± 23	15 ± 5/12 ± 3	3,484 ± 2,072 mGy·cm^2^/3,168 ± 2,346 mGy·cm^2^	100/100	3.5/5.2	77.2/80.7
Abdullah (21)	76.5 (59–100)/141 (111.5–165.5)	23.3 (19–31.3)/10.6 (5.75–16.2)	NR/NR	100/100	2/1	NR/NR

**Table 6:** Reported complications by treatment group.

Study	PFA group	vhpsd group
Campanelli (11)	vascular access complications	post-procedural pericarditis; 1 major complication—post-procedural stroke
Cardinali (12)	minor complications	minor complications
Dello Russo (13)	3 major vascular complications; 1 minor; 1 pericarditis; 1 femoral hematoma	5 pericarditis; 1 major vascular complications
Orban (14)	1 groin hematoma; 1 ST elevation (as reported); 1 femoral artery fistula	1 groin hematoma
Popa (15)	5 groin complications; 1 stroke	4 groin hematoma; 1 stroke; 1 stroke
Regany-Closa (16)	NR	NR
Santos (17)	1 acute cardiac tamponade	None reported
Soubh (18)	None reported	1 stroke
Weyand (19)	1 transient hemolysis	2 minor vascular access complications; 2 pseudoaneurysms
Wörmann (20)	2 cardiac tamponade	2 cardiac tamponade; 1 pulmonary vein stenosis
Abdullah (21)	1 cardiac tamponade; 1 pericardial effusion with slow ventricular rate	1 cardiac tamponade

The table labeled “NR” cell, on behalf of the corresponding original research in the original not the baseline indicators, we are unable to obtain related data from open literature.

Because baseline imbalances may confound efficacy and safety comparisons, we first assessed the comparability of the PFA and vHPSD groups at enrollment. Across all examined variables, the two groups were broadly balanced. The mean age was slightly lower in the PFA group (*MD* = −1.43 years, 95%*CI*: −2.49 to −0.36), and the left atrial diameter was slightly larger in the PFA group (*MD* = 1.42 mm, 95%*CI*: 0.79 to 2.06). However, the absolute differences were small, and the more integrative left atrial volume index showed no significant between-group difference. Sex, BMI, type of atrial fibrillation, hypertension, diabetes, coronary artery disease, LVEF, and CHA_2_DS_2_–VASc score did not differ significantly between groups. The pooled results and sensitivity analyses for each baseline variable are detailed in the Supplementary Materials (Supplementary Figures S1, S2).

### 3.3 Risk of bias assessment results

**Figure 2:**
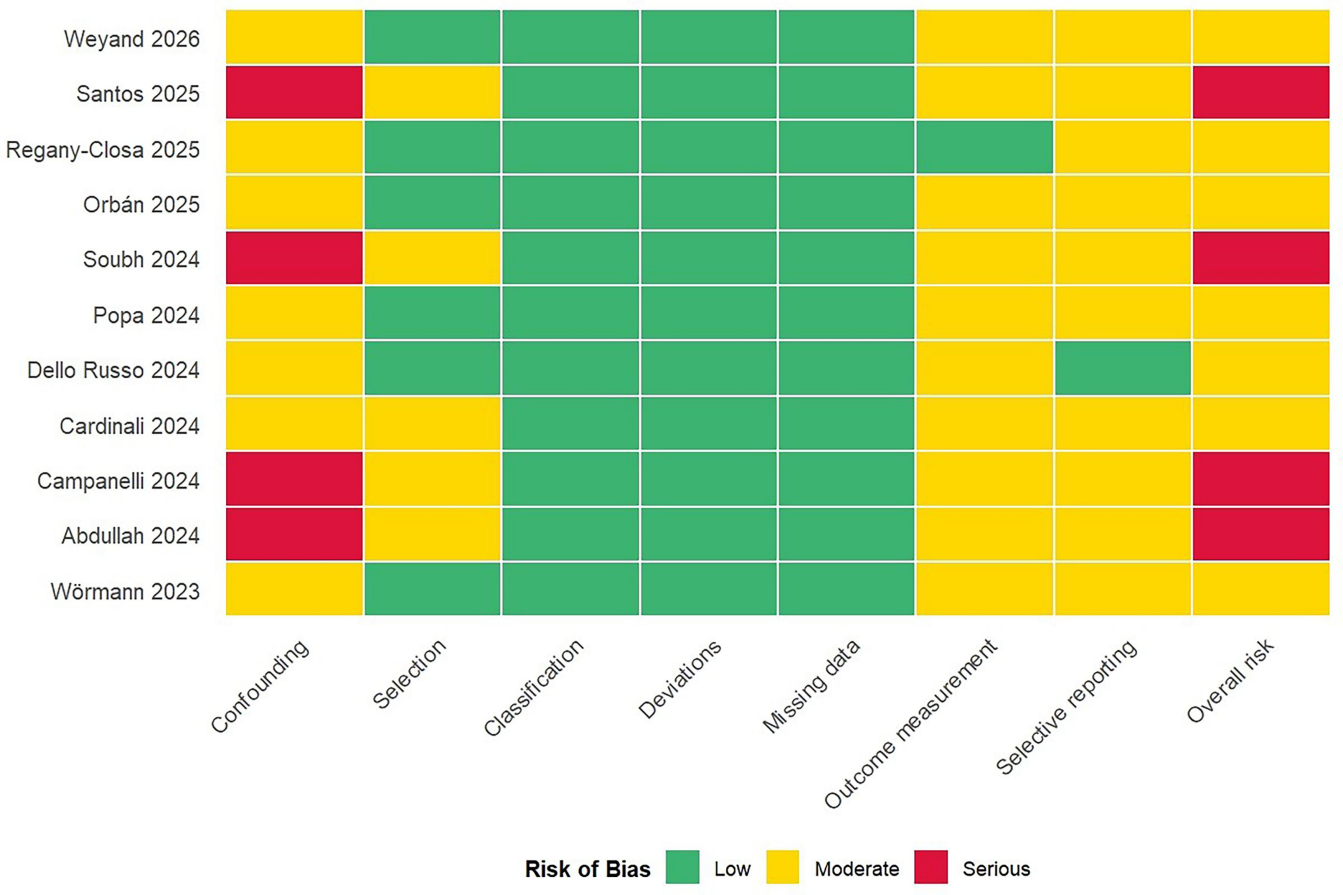
Quality appraisal of the included studies using ROBINS-I.

The 11 observational studies were evaluated via the ROBINS-I tool (Figure 2) and the Newcastle-Ottawa Scale (NOS) (Table 7). The ROBINS-I assessment identified 4 studies at high risk and 7 at moderate risk, while the NOS rated 10 studies as high quality and 1 as moderate quality. Because NOS primarily appraises reporting completeness and design, whereas ROBINS-I focuses on the internal validity of effect estimates, this partial divergence is to be expected. Overall, the quality of the included studies was deemed acceptable for the subsequent analyses.

**Table 7:** Quality assessment using the Newcastle-Ottawa scale.

Study	Selection (0–4 points)	Comparability (0–2)	Outcome (0–3)	Total Score (0–9)	Grade of quality
Wörmann (20)	4	2	3	9/9	High
Abdullah (21)	4	0	2	6/9	Medium
Campanelli (11)	4	1	3	8/9	High
Cardinali (12)	4	2	3	9/9	High
Dello Russo (13)	4	2	3	9/9	High
Popa (15)	4	2	3	9/9	High
Soubh (18)	4	1	3	8/9	High
Orbán (14)	4	2	3	9/9	High
Regany-Closa (16)	4	2	3	9/9	High
Santos (17)	4	1	3	8/9	High
Weyand (19)	4	2	3	9/9	High

### 3.4 Results of meta-analysis

#### 3.4.1 Effectiveness analysis

##### 3.4.1.1 Freedom from arrhythmia during follow-up

###### 3.4.1.1.1 Main analysis results

**Figure 3:**
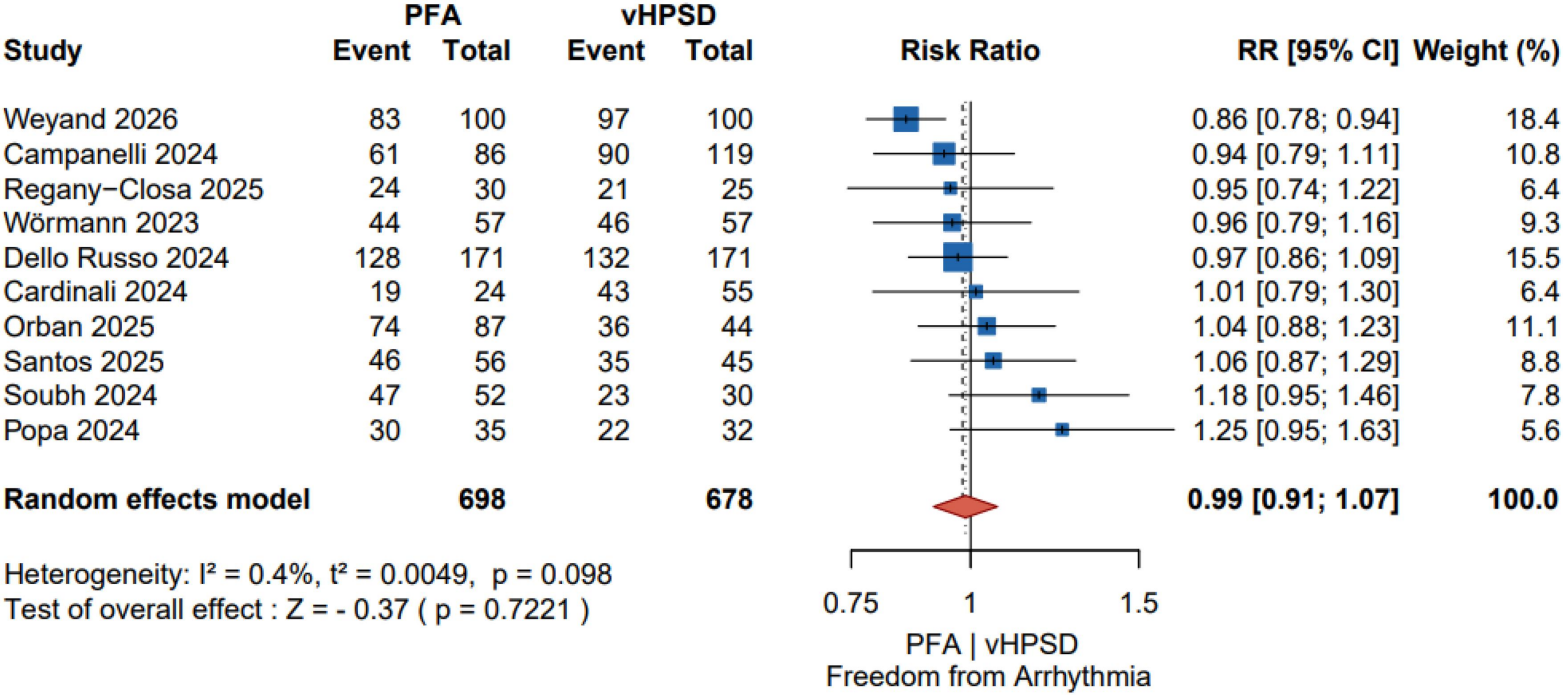
Forest plot of the main analysis results.

This outcome measure was reported in 10 studies involving 1,376 patients with AF, including 698 patients in the PFA group and 678 patients in the vHPSD group. The combined results showed that there was no significant difference in the arrhythmia free rate between the PFA group and the vHPSD group (*RR* = 0.99, 95%CI: 0.91 to 1.07, *P* = 0.722, Figure 3), indicating that the efficacy of the two ablation methods may be comparable. There was no significant heterogeneity among the included studies (*I*^2^ = 0.4%, *P* = 0.098), suggesting that the results were highly consistent among the studies. This consistency was further confirmed by the results of individual studies: the RRS of all included studies ranged from 0.86 to 1.25, and the 95% confidence intervals of all studies crossed 1, further indicating that the combined results of this study were highly robust.

###### 3.4.1.1.2 Subgroup analysis

**Figure 4:**
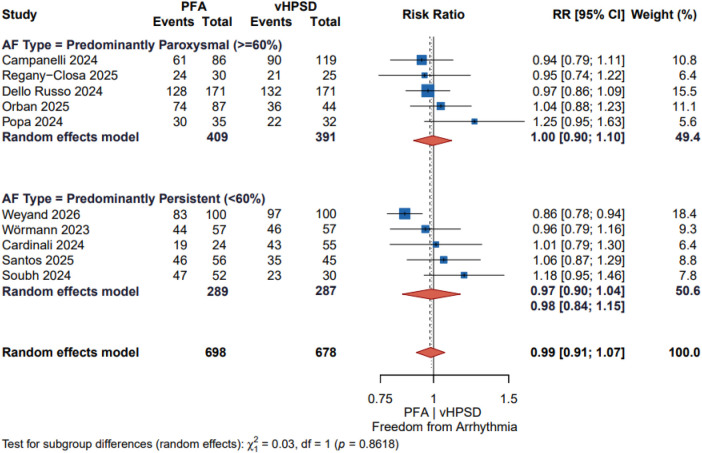
Forest plot of the subgroup analysis.

The results of subgroup analysis showed that the results of both subgroups were highly consistent with the main analysis: subgroup dominated by paroxysmal atrial fibrillation: Five studies involving 845 patients were included (409 patients in PFA group and 391 patients in vHPSD group, Figure 4). The combined effect size showed that there was no significant difference in the rate of arrhythmia free after surgery between PFA and vHPSD (*RR* = 1.00, 95%CI: 0.90 to 1.10). In the subgroup of persistent AF, 5 studies involving 576 patients (289 in PFA group and 287 in vHPSD group) were included. The combined effect size showed that the efficacy of the two ablation methods was similar (*RR* = 0.98, 95%CI: 0.84 to 1.15). There was no significant interaction between the two ablation methods (*χ*^2^ = 0.03, df = 1, *P* = 0.8618), suggesting that the conclusion that PFA and vHPSD may have the same efficacy has good consistency and extrapolation in different AF types.

###### 3.4.1.1.3 Sources of heterogeneity (meta-regression)

To explore potential sources of heterogeneity among the included studies, we performed univariate meta-regression analyses evaluating six prespecified covariates: proportion of paroxysmal atrial fibrillation, mean age, proportion of males, left ventricular ejection fraction, CHA2DS2-VASc score, and duration of follow-up.

The results showed that none of the included covariates were statistically associated with the study effect size, and the exponential transformation value of regression coefficients suggested that there was only a small non-significant fluctuation in the risk ratio of freedom from arrhythmia during follow-up for each 1-unit increase in each covariate.

**Figure 5:**
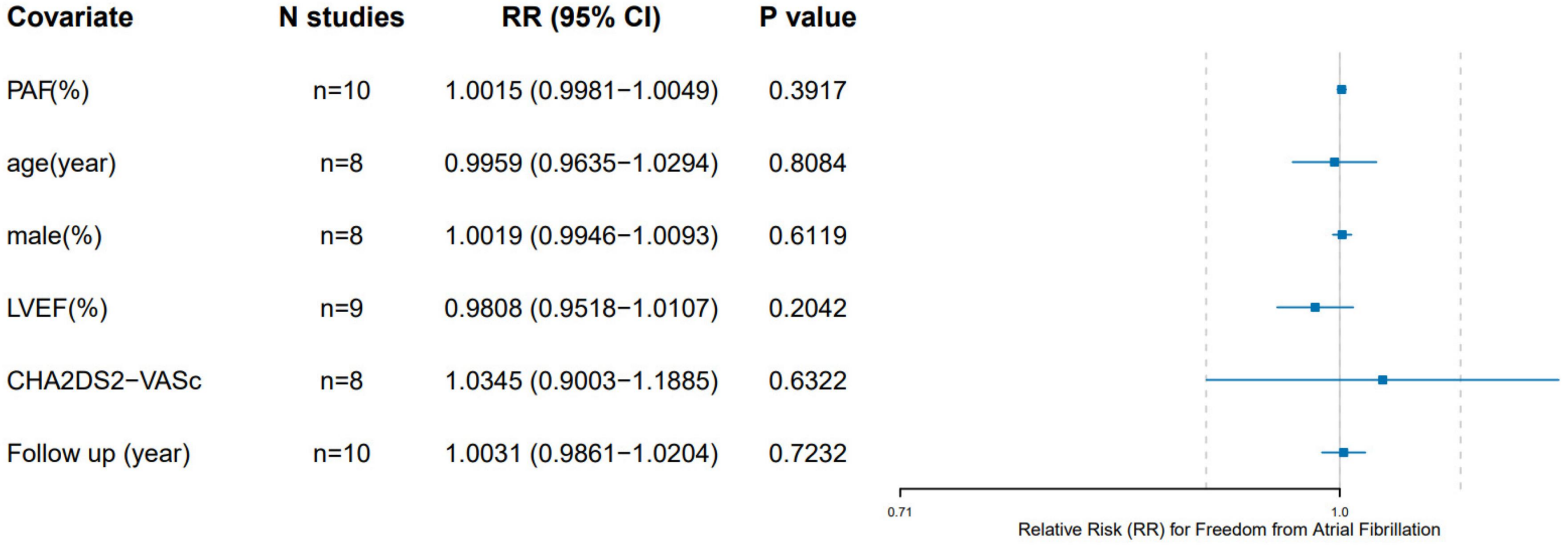
Meta-regression.

This finding suggests that the observed heterogeneity across the included studies was not due to differences in these common baseline clinical characteristics (Figure 5).

###### 3.4.1.1.4 Sensitivity analysis

Sensitivity analysis supported the robustness of the primary analysis for Freedom from arrhythmia during follow-up. When alternative pooling models were applied, the pooled *RRs* ranged from 0.981 to 0.988, and all 95%CIs crossed the null value, indicating no significant difference between the two groups (Supplementary Table S2). After excluding studies published only as conference abstracts, the pooled estimate remained consistent with the main analysis (*RR* = 0.998, 95%CI: 0.903 to 1.102, *P* = 0.955, Supplementary Figure S3). Similarly, using *OR* instead of *RR* did not materially change the result (*OR* = 0.962, 95%CI: 0.601 to 1.541, *P* = 0.858, Supplementary Figure S4). Leave-one-out analysis showed that the pooled RR remained stable at approximately 0.98 to 0.99 (Supplementary Figure S5), suggesting that no single study had a disproportionate influence on the pooled estimate.

###### 3.4.1.1.5 Publication bias

**Figure 6:**
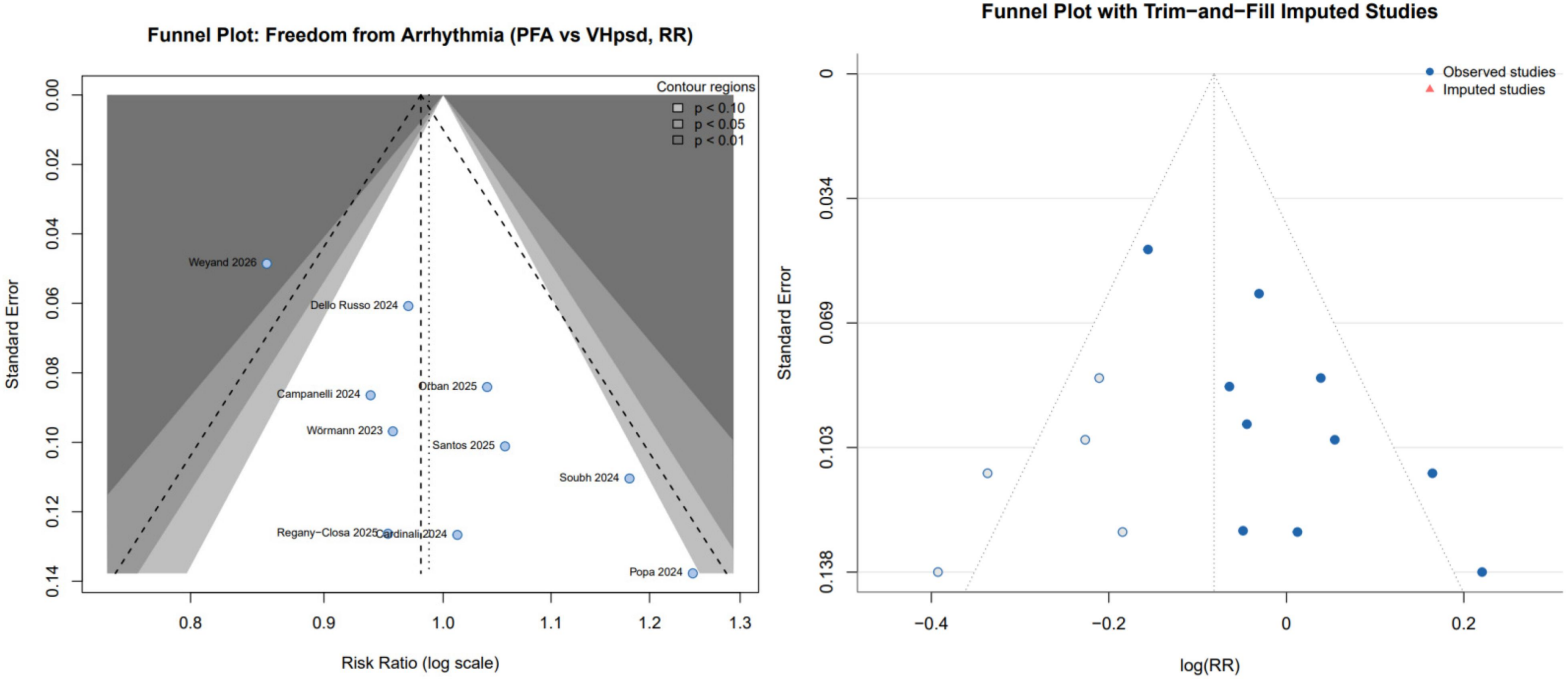
Funnel plot.

The funnel plot demonstrated a roughly symmetrical distribution of studies, albeit with a slight asymmetry. Begg's test yielded a *P* value of 0.089, while Egger's test yielded a *P* value of 0.0086, the latter suggesting potential publication bias, possibly attributable to the non-publication of small studies with negative findings. We further applied the Duval and Tweedie trim-and-fill method for correction, which estimated and imputed five potentially missing studies on the left side of the funnel plot. After correction, the pooled *RR* changed from 0.988 (95%CI: 0.919 to 1.061, *P* = 0.747) to 0.922 (95%CI: 0.854 to 0.995, *P* = 0.0356). The direction of the effect was not reversed, and the absolute difference in freedom from arrhythmia between the two groups remained minimal and clinically insignificant. These findings indicate that even if a certain degree of publication bias exists, its impact on the pooled effect estimate is limited, and the core conclusion of this study has not been substantially altered, confirming the robustness of the results (Figure 6).

##### 3.4.1.2 Immediate pulmonary vein isolation rate

###### 3.4.1.2.1 Main analysis results

**Figure 7:**
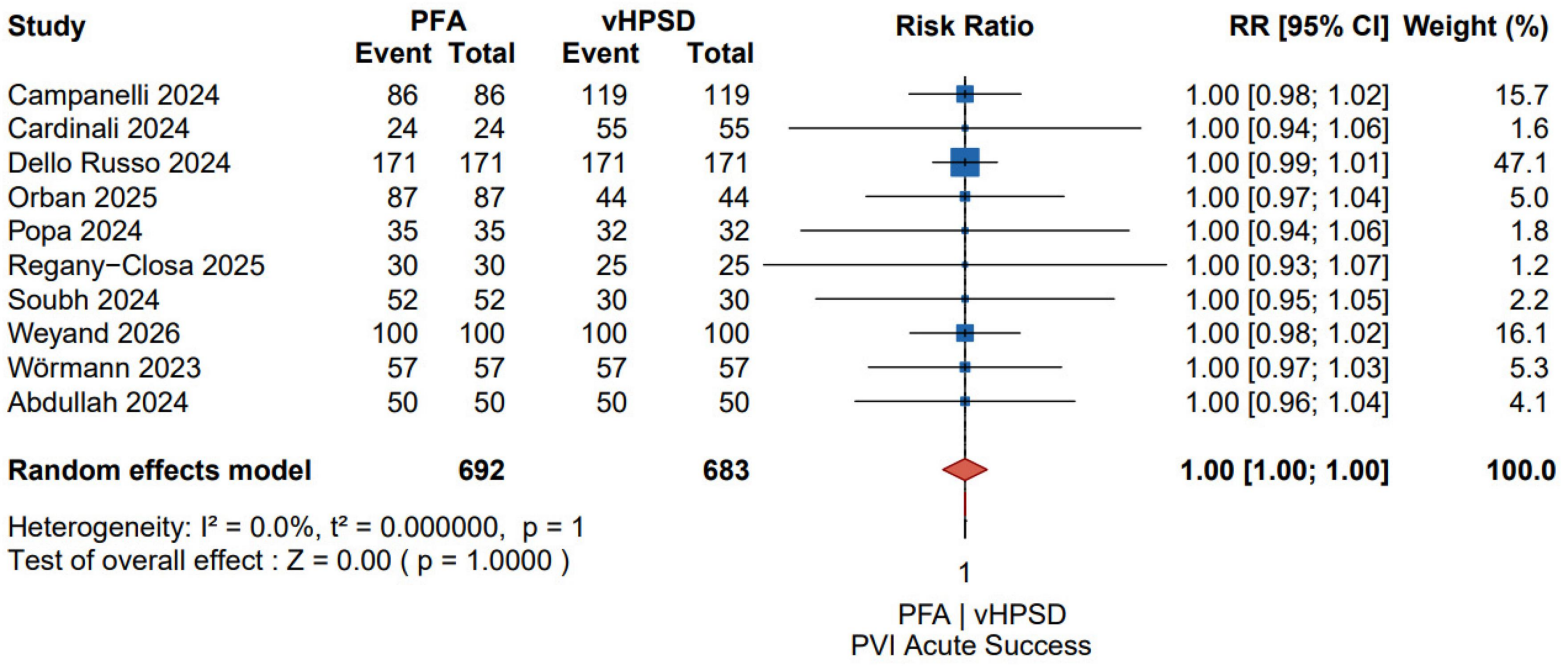
Forest plot of the main analysis results.

This outcome measure was reported in 10 studies involving 1,375 patients with AF, including 692 patients in the PFA group and 683 patients in the vHPSD group. The combined results showed that there was no significant difference in the immediate success rate of pulmonary vein isolation (PVI) between the PFA group and the vHPSD group (*RR* = 1.00, 95%CI: 1.00 to 1.00, *P* = 1.000, Figure 7), which meant that the immediate PVI success rate of the two ablation methods was completely consistent. There was no statistical heterogeneity among the included studies (*I^2^* = 0.0%, *P* = 1.000), suggesting that the results were highly consistent among the studies. The results of the individual studies further verified this consistency: the RR value of all included studies was 1.00, and the 95% confidence interval of all studies crossed 1, further indicating that the combined results of this study were extremely robust.

###### 3.4.1.2.2 Subgroup analysis

**Figure 8:**
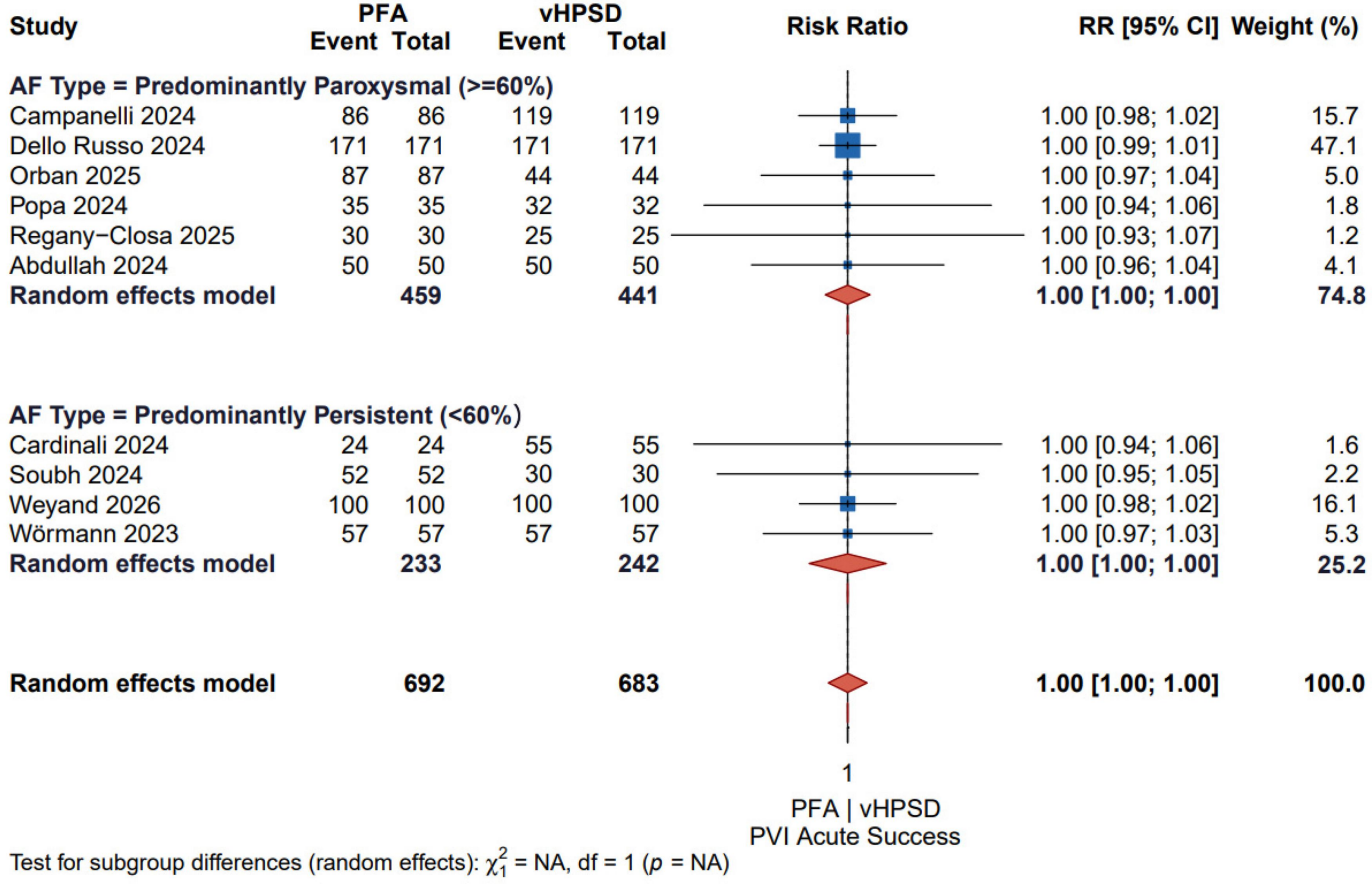
Forest plot of the subgroup analysis.

The results of subgroup analysis showed that the results of both subgroups were highly consistent with the main analysis: subgroup dominated by paroxysmal atrial fibrillation: Six studies involving 900 patients (459 in the PFA group and 441 in the vHPSD group) were included (Figure 8). The pooled effect size results showed that there was no significant difference in the success rate of immediate pulmonary vein isolation (PVI) between PFA and vHPSD (*RR* = 1.00, 95%CI: 1.00 to 1.00); In the subgroup of persistent AF, 4 studies with 475 patients (233 in PFA group and 242 in vHPSD group) were included. The combined effect size showed that the immediate isolation success rate of the two ablation methods was also completely consistent (*RR* = 1.00, 95%CI: 1.00 to 1.00). The results of difference test between subgroups showed that the effect size of the two subgroups was completely consistent, and no significant interaction effect of AF type was observed on the difference in the immediate isolation success rate of the two ablation methods, suggesting that the conclusion that the PFA and vHPSD are completely consistent with the immediate isolation success rate of the two ablation methods has good consistency and extrapolation in different AF types.

###### 3.4.1.2.3 Sources of heterogeneity (meta-regression)

To explore potential sources of heterogeneity among the included studies, we performed univariate meta-regression analyses evaluating six prespecified covariates: proportion of paroxysmal atrial fibrillation, mean age, proportion of males, left ventricular ejection fraction, CHA2DS2-VASc score, and duration of follow-up.

**Figure 9:**
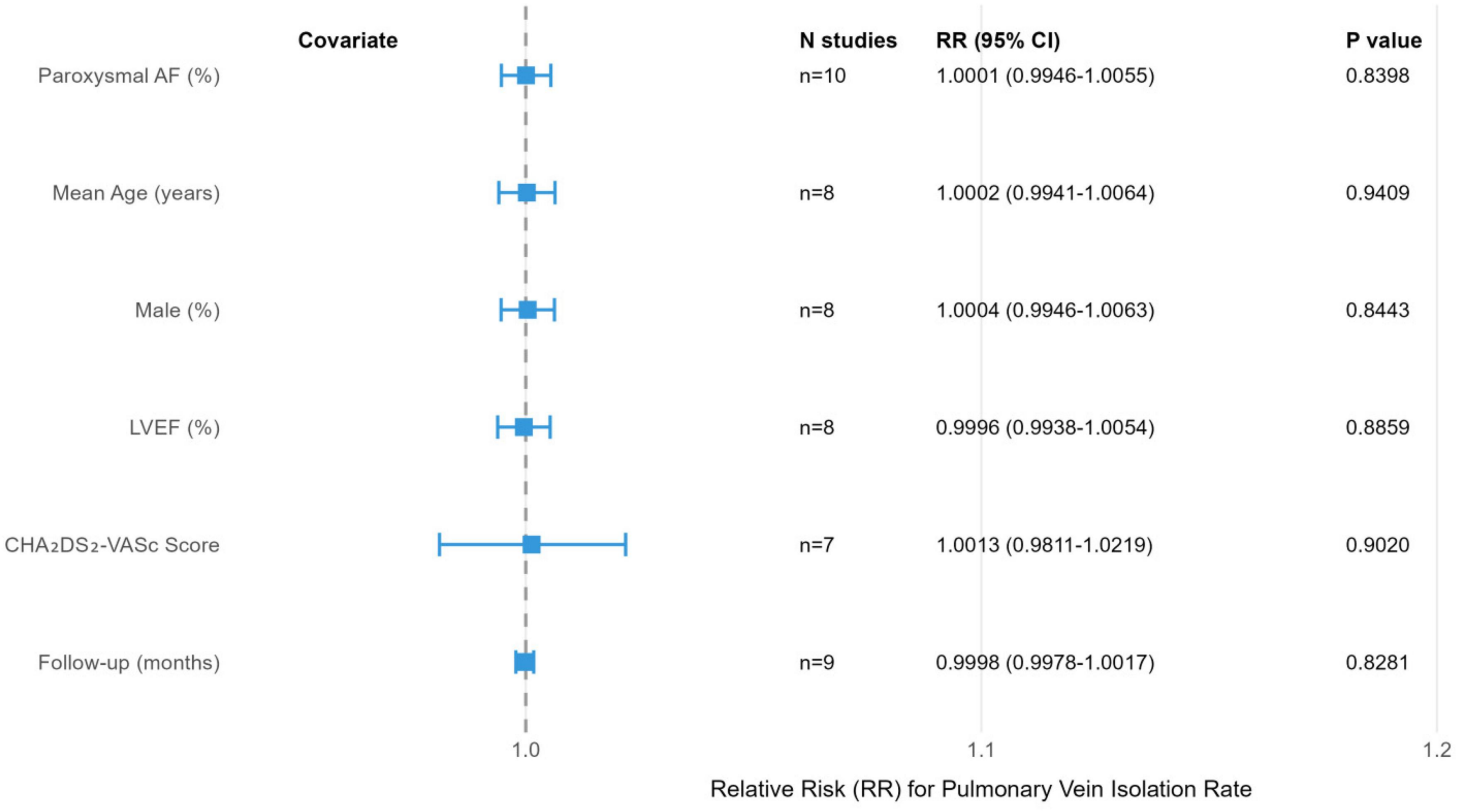
Meta-regression.

None of the included covariates was statistically associated with the study effect size, and regression coefficients suggested that the effect size of the immediate PVI rate fluctuated only minimally and non-significantly with an increase of one unit in each covariate. This finding suggests that the very low heterogeneity observed across the included studies was not due to differences in these common baseline clinical characteristics (Figure 9).

###### 3.4.1.2.4 Sensitivity analysis

Sensitivity analyses confirmed the robustness of the primary analysis for the immediate PVI success rate. Across alternative pooling models, the pooled *RRs* were essentially unchanged, ranging from 1.0000 to 1.0003, with 95%CIs including the null value (Supplementary Table S2). After excluding studies published only as conference abstracts, the result remained identical to the main analysis (*RR* = 1.00, 95%*CI*: 1.00 to 1.00, *P* = 1.0000, Supplementary Figure S3). Reanalysis using *OR* instead of *RR* also showed no significant difference between the two groups (Supplementary Figure S4). In the leave-one-out analysis, the pooled *RR* remained stable at approximately 0.99 to 1.00 after sequential exclusion of individual studies, indicating that no single study materially influenced the pooled estimate (Supplementary Figure S5).

###### 3.4.1.2.5 Publication bias

**Figure 10:**
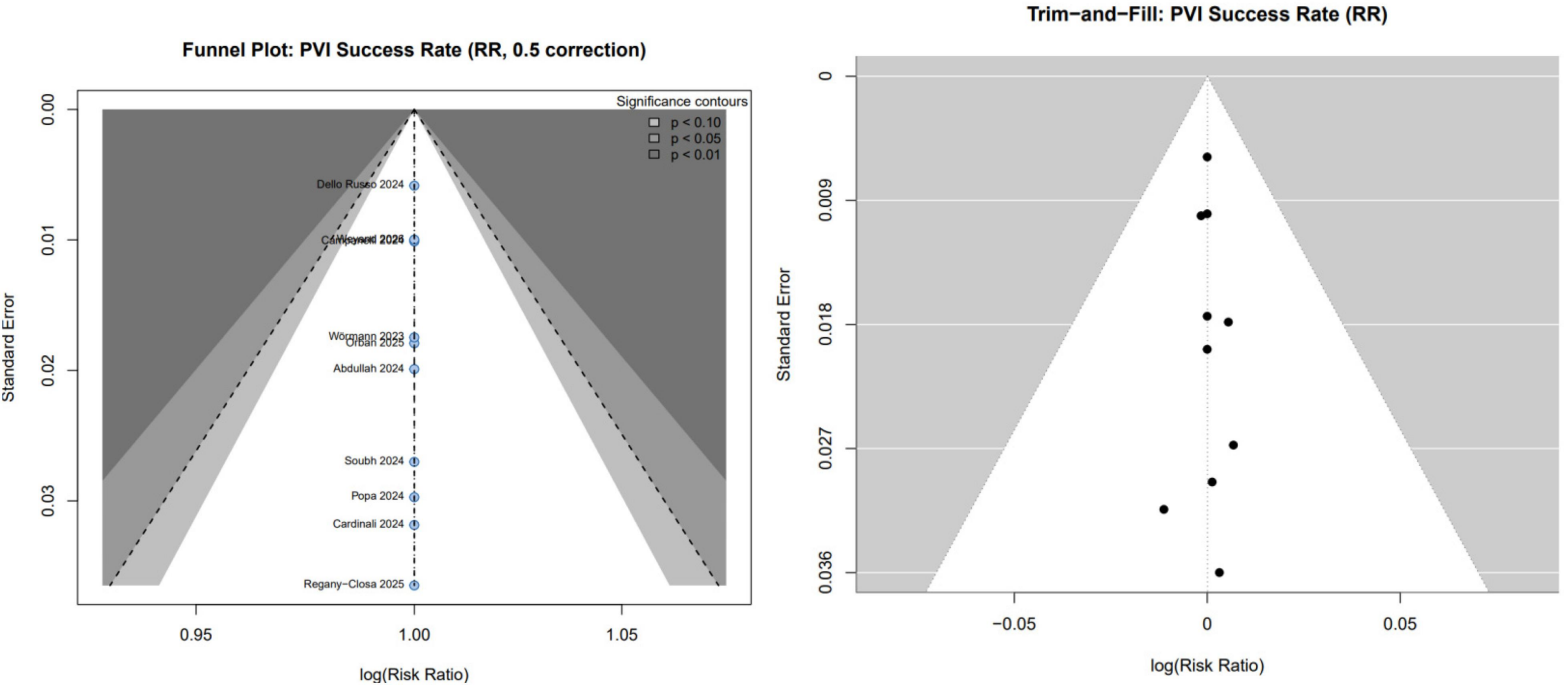
Funnel plot.

On the funnel plot, all studies fell precisely on the central line of no effect (*RR* = 1), with a perfectly symmetrical distribution. Both Begg's test and Egger's test could not be performed because there was no variability in the effect estimates (statistic = NA). Furthermore, the Duval and Tweedie trim-and-fill method did not estimate any potentially missing studies on either side of the funnel plot. The pooled effect estimate remained identical before and after imputation, with a pooled *RR* of 1.00 (95%CI: 1.00 to 1.00, *P* = 1.000). Therefore, no evidence of publication bias was detected in this study, and bias correction did not cause any alteration in the pooled effect estimate or the direction of effect, confirming that the core conclusion is of extremely high reliability (Figure 10).

#### 3.4.2 Safety analysis

##### 3.4.2.1 Total incidence of surgery-related complications

###### 3.4.2.1.1 Main analysis results

**Figure 11:**
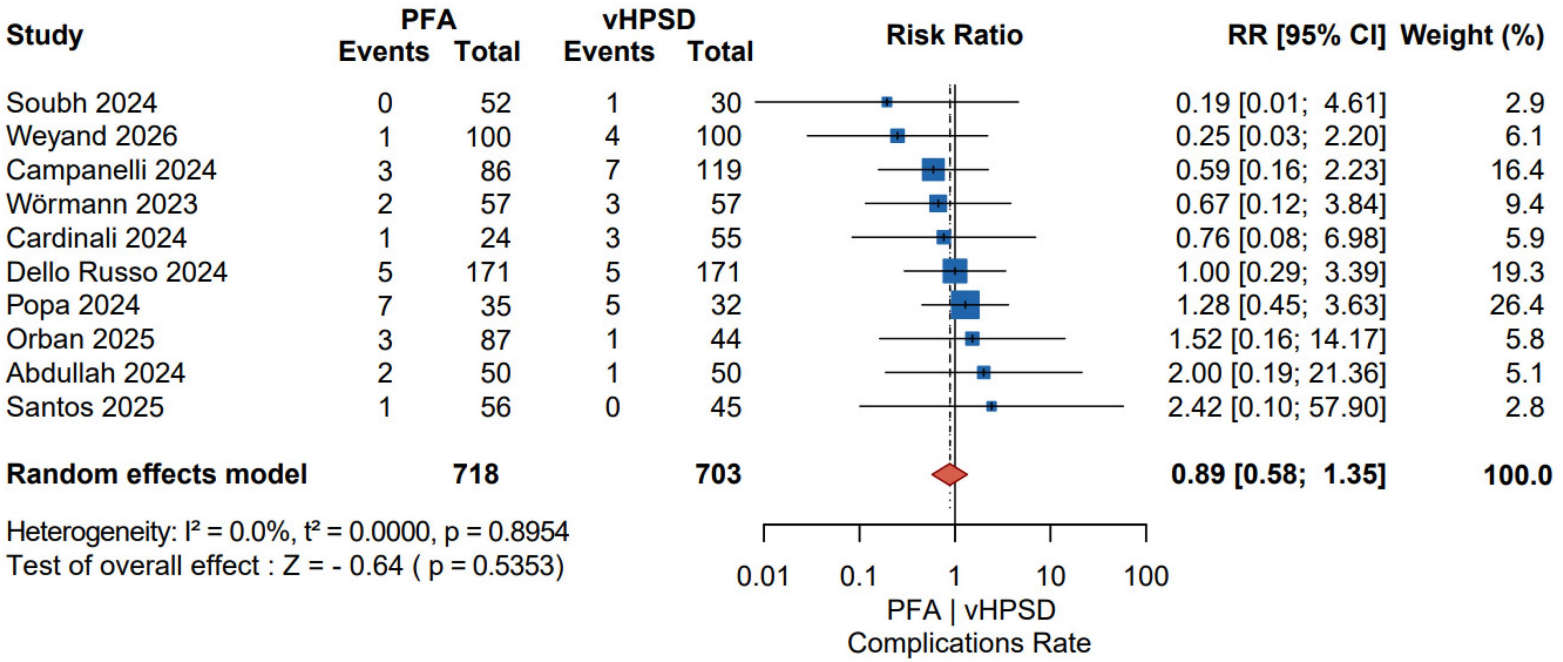
Forest plot of the main analysis results.

This outcome measure was reported in 10 studies involving 1,421 patients with AF, including 718 in the PFA group and 703 in the vHPSD group. The combined results showed that there was no significant difference in the incidence of postoperative complications between the PFA group and the vHPSD group (*RR* = 0.89, 95%CI: 0.58 to 1.35, *P* = 0.5353, Figure 11), indicating that the perioperative safety of the two ablation methods may be comparable. There was no statistical heterogeneity among the included studies (*I^2^* = 0.0%, *P* = 0.8954), suggesting that the results were highly consistent among the studies. This consistency was further confirmed by the results of individual studies: the *RRS* of all included studies ranged from 0.19 to 2.42, and the 95% confidence intervals of all studies were across 1, further indicating that the combined results of this study were highly robust.

###### 3.4.2.1.2 Subgroup analysis

**Figure 12:**
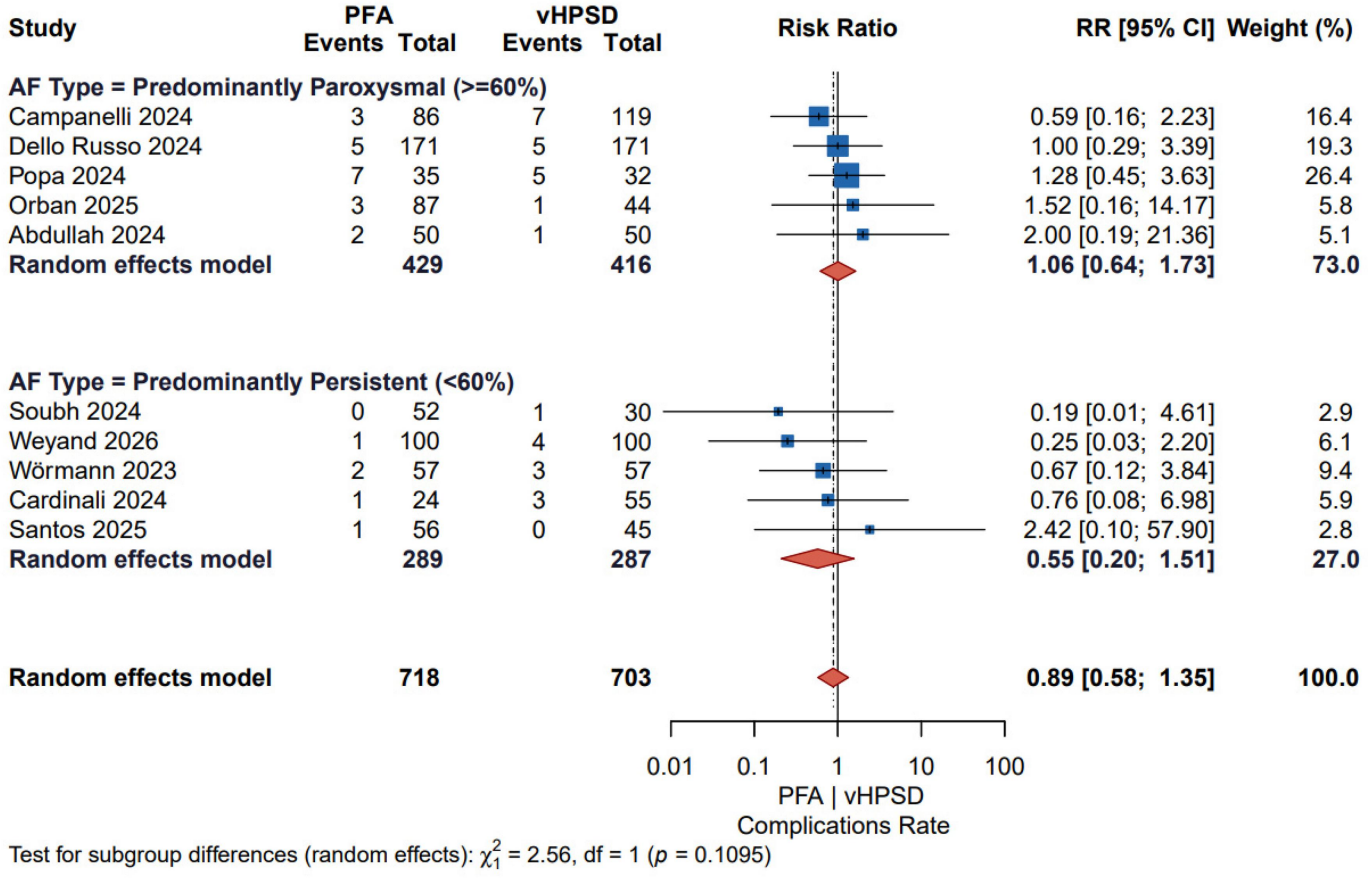
Forest plot of the subgroup analysis.

The results of subgroup analysis showed that the results of both subgroups were highly consistent with the main analysis: subgroup dominated by paroxysmal atrial fibrillation: Five studies involving 845 patients (429 in the PFA group and 416 in the vHPSD group) were included. The combined effect size showed that there was no significant difference in the incidence of postoperative complications between PFA and vHPSD (*RR* = 1.06, 95%CI: 0.64 to 1.73, Figure 12). In the subgroup of persistent AF, 5 studies involving 576 patients were included (289 patients in PFA group and 287 patients in vHPSD group). The combined effect size showed that the perioperative safety of the two ablation methods was similar (*RR* = 0.55, 95%CI: 0.20 to 1.51). There was no significant interaction effect of atrial fibrillation type on the incidence of complications between the two ablation methods (*χ^2^* = 2.56, *df* = 1, *P* = 0.1095), suggesting that the perioperative safety of PFA and vHPSD may be equivalent. It has good consistency and extrapolation in patients with different types of AF.

###### 3.4.2.1.3 Sources of heterogeneity (meta-regression)

**Figure 13:**
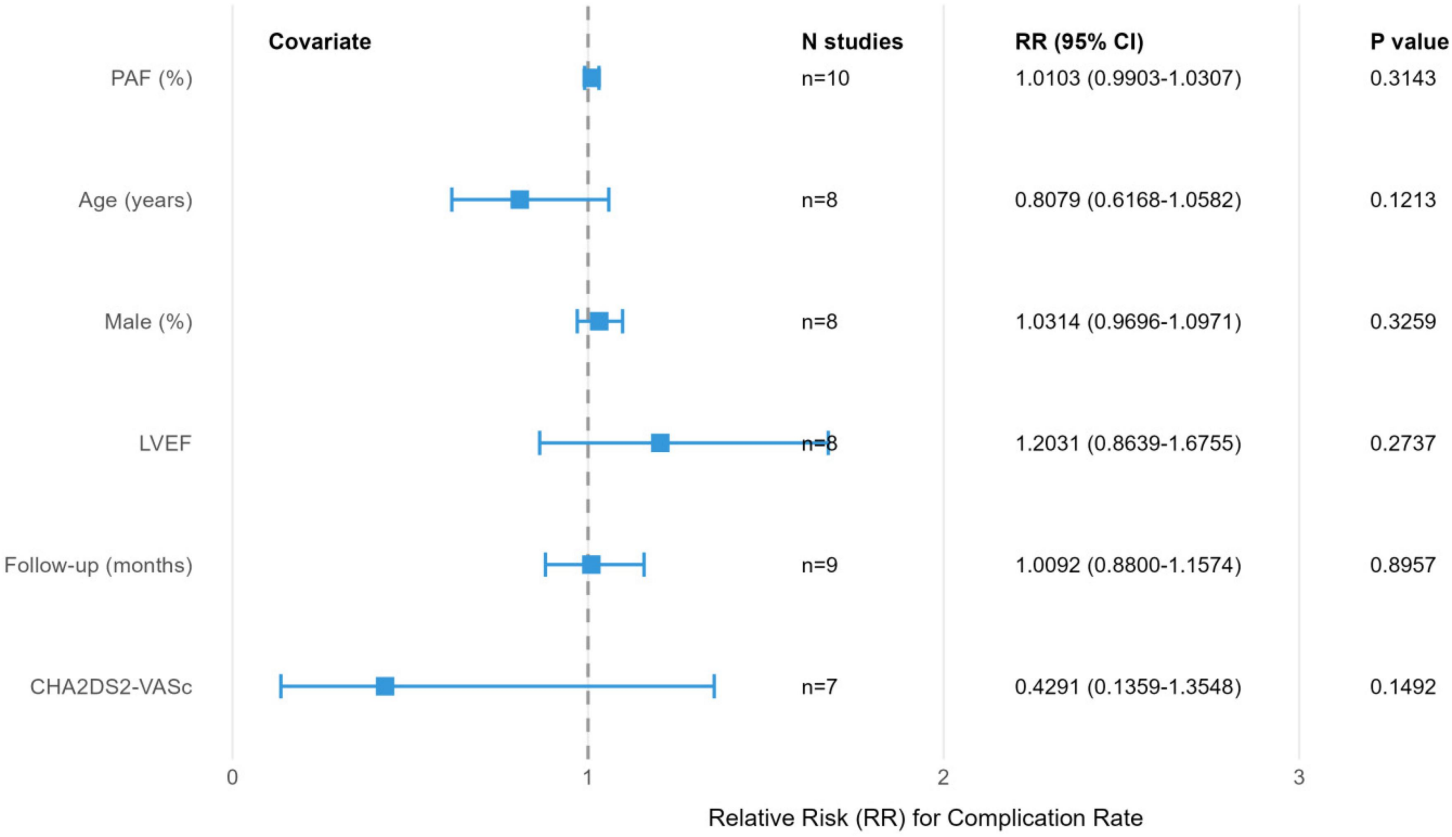
Meta-regression.

To explore potential sources of heterogeneity among the included studies, we performed univariate meta-regression analyses evaluating six prespecified covariates: proportion of paroxysmal atrial fibrillation, mean age, proportion of males, left ventricular ejection fraction, CHA2DS2-VASc score, and duration of follow-up. The results showed that none of the included covariates was statistically associated with the study effect size, and the exponential transformation value of regression coefficients suggested that there was only a small non-significant fluctuation in the risk ratio of postoperative complications with an increase of one unit in each covariate (Figure 13).

###### 3.4.2.1.4 Sensitivity analysis

Sensitivity analyses supported the robustness of the primary analysis for postoperative complications. Across alternative pooling models, the pooled *RRs* ranged from 0.85 to 0.89, and all 95%CIs included the null value (Supplementary Table S2), indicating no significant difference between the two groups. After excluding studies published only as conference abstracts, the result remained nonsignificant (*RR* = 0.93, 95%CI: 0.51 to 1.67, *P* = 0.765, Supplementary Figure S3). Reanalysis using *OR* instead of *RR* yielded a similar conclusion (*OR* = 0.86, 95%CI: 0.55 to 1.36, *P* = 0.487, Supplementary Figure S4). In the leave-one-out analysis, exclusion of individual studies did not alter the statistical interpretation of the result, with pooled *RRs* remaining nonsignificant, suggesting that no single study had a decisive influence on the overall conclusion (Supplementary Figure S5).

###### 3.4.2.1.5 Publication bias

**Figure 14:**
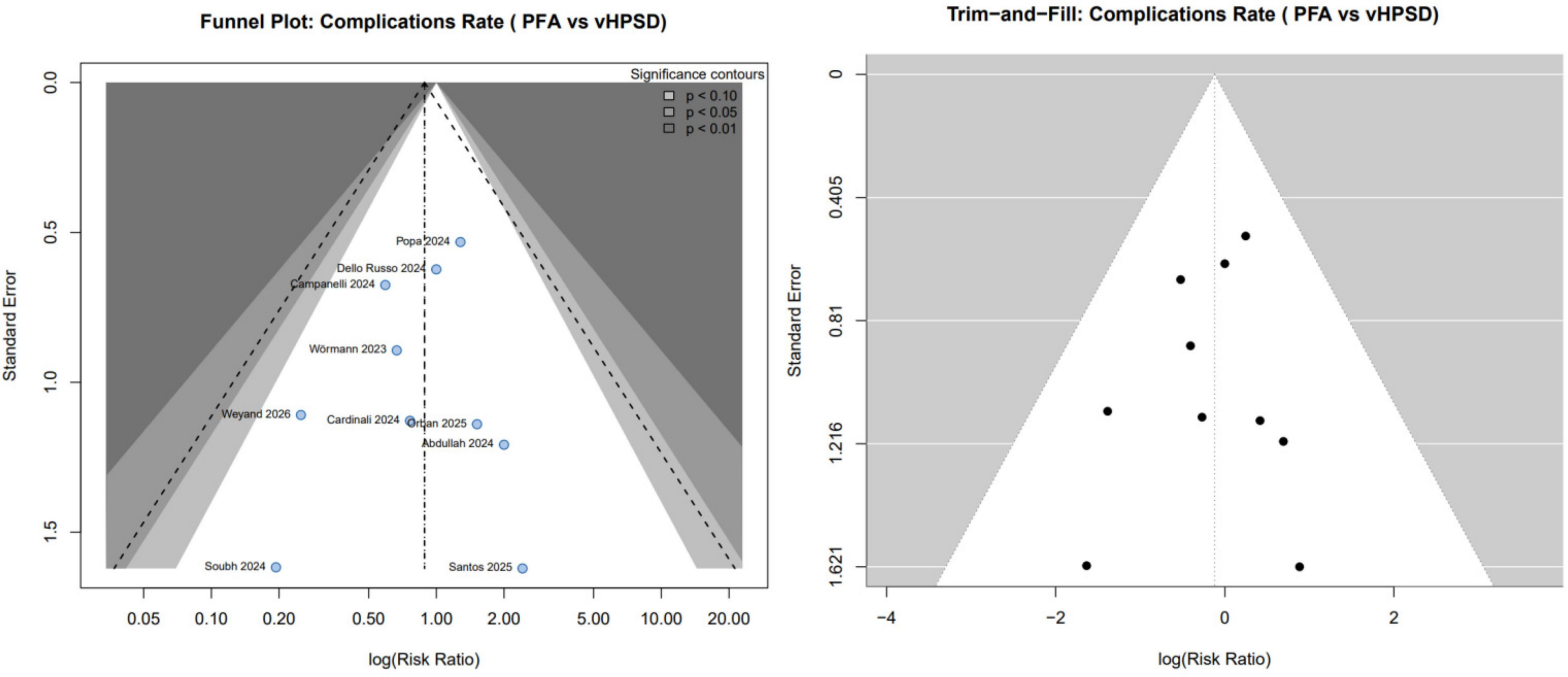
funnel plot.

The funnel plot showed a roughly symmetrical distribution of studies with no obvious asymmetry. Begg's test yielded *P* = 0.721 and Egger's test yielded *P* = 0.892, with neither test detecting significant publication bias. Further application of the Duval and Tweedie trim-and-fill method did not estimate any potentially missing studies in the funnel plot, and the pooled *RR* was identical before and after imputation (*RR* = 0.89, 95%CI: 0.52 to 1.51, *P* = 0.535), with both the effect estimate and direction remaining completely consistent. The results indicate that although the postoperative complication rate was slightly lower in the PFA group, the difference between the two groups was not statistically significant. Overall, no publication bias was detected in this study, and bias correction caused no change in the pooled effect estimate, confirming that the core conclusion remains highly reliable (Figure 14).

##### 3.4.2.2 Incidence of single complication

###### 3.4.2.2.1 Main analysis results

**Figure 15:**
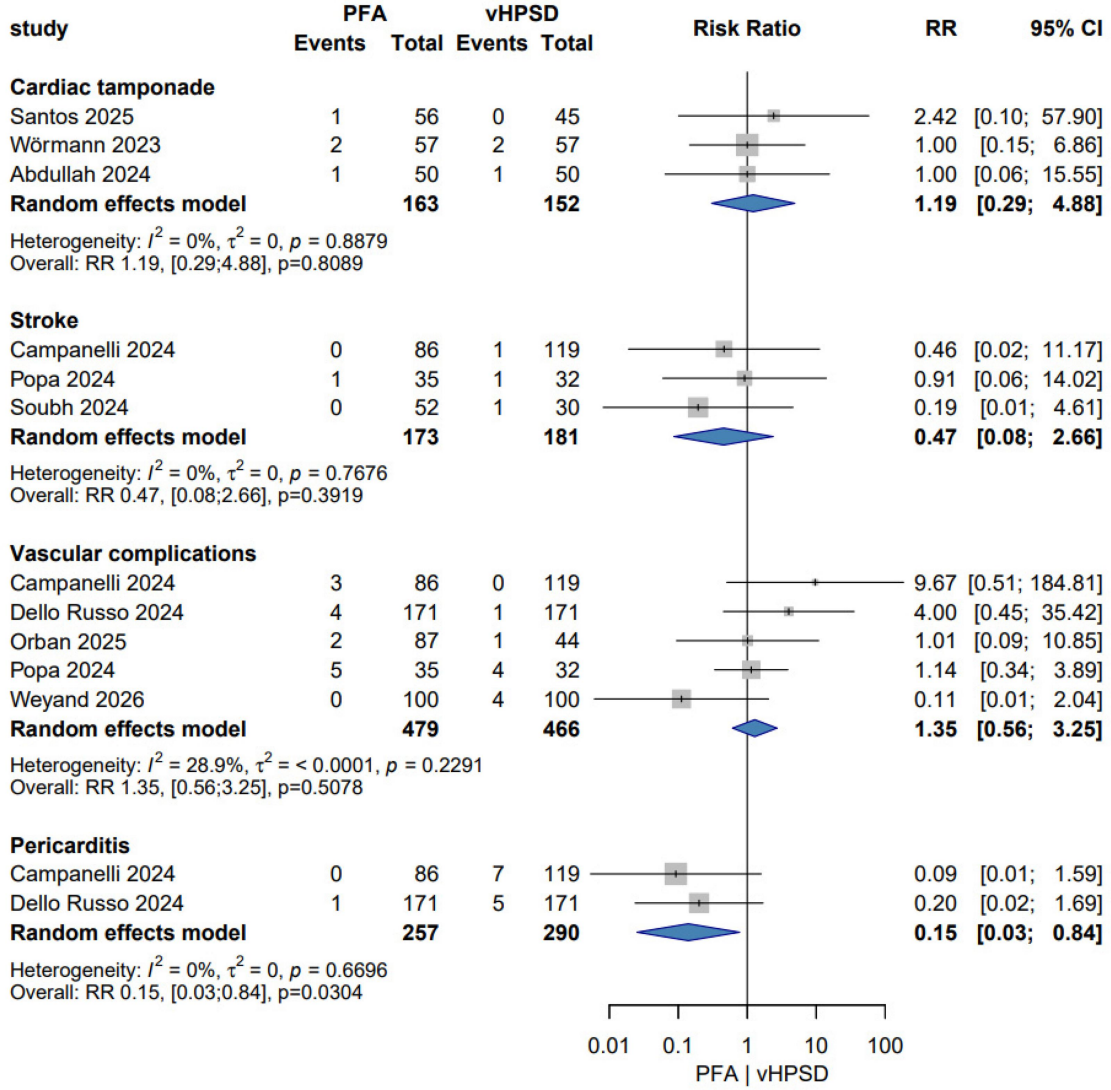
Forest plot of the main analysis results.

To further refine the assessment of perioperative safety differences between the two ablation techniques, we performed a subsubgroup analysis of postoperative complications to assess differences in the incidence of four common perioperative adverse events (Figure 15): cardiac tamponade, stroke, vascular complications, and pericarditis, with the following results:

1. Cardiac tamponade: Three studies involving 315 patients (PFA group 163, vHPSD group 152) were included. The pooled results showed that there was no significant difference in the incidence of cardiac tamponade between PFA group and vHPSD group (*RR* = 1.19, 95%CI: 0.29 to 4.88, *P* = 0.809). There was no statistical heterogeneity among the included studies (*I^2^* = 0.0%, *P* = 0.888), suggesting that the results were highly consistent among the studies.
2. Stroke: Three studies with 346 patients (173 in PFA group and 173 in vHPSD group) were included. The pooled results showed that there was no significant difference in the incidence of stroke between PFA group and vHPSD group (*RR* = 0.47, 95%CI: 0.08 to 2.66, *P* = 0.392). There was no statistical heterogeneity among the included studies (*I^2^* = 0.0%, *P* = 0.768), suggesting that the results were consistent among the studies.
3. Vascular complications: Five studies involving 945 patients (479 patients in PFA group and 466 patients in vHPSD group) were included. The pooled results showed that there was no significant difference in the incidence of vascular complications between the PFA group and the vHPSD group (*RR* = 1.35, 95%CI: 0.56 to 3.25, *P* = 0.508). There was a low degree of heterogeneity among the included studies (*I^2^* = 28.9%, *P* = 0.229), but it did not materially affect the results.
4. Pericarditis: Two studies involving 547 patients were included (257 in PFA group and 290 in vHPSD group). The pooled results showed that the incidence of pericarditis in the PFA group was significantly lower than that in the vHPSD group (*RR* = 0.15, 95%CI: 0.03 to 0.84, *P* = 0.030), suggesting that PFA technique may have some advantages in reducing the risk of perioperative pericarditis. There was no statistical heterogeneity among the included studies (*I^2^* = 0.0%, *P* = 0.670), suggesting that the results were consistent among the studies.

###### 3.4.2.2.2 Sensitivity analysis

Sensitivity analyses were performed to assess the robustness of the findings for individual complications. Across alternative pooling models, the pooled *RRs* ranged from 0.85 to 0.89, and all 95%CIs included the null value (Supplementary Table S2), indicating no significant difference in the overall incidence of postoperative complications between the two groups. Reanalysis using *OR* instead of *RR* yielded consistent findings for individual complication outcomes: no significant differences were observed for cardiac tamponade, vascular complications, or pericarditis, while the finding for stroke remained stable without reversal of direction or statistical significance (Supplementary Figure S4). Leave-one-out analyses also showed no material changes in the pooled estimates or statistical interpretation after sequential exclusion of individual studies, suggesting that no single study had a disproportionate influence on the subgroup results (Supplementary Figure S5).

###### 3.4.2.2.3 Publication bias

**Figure 16:**
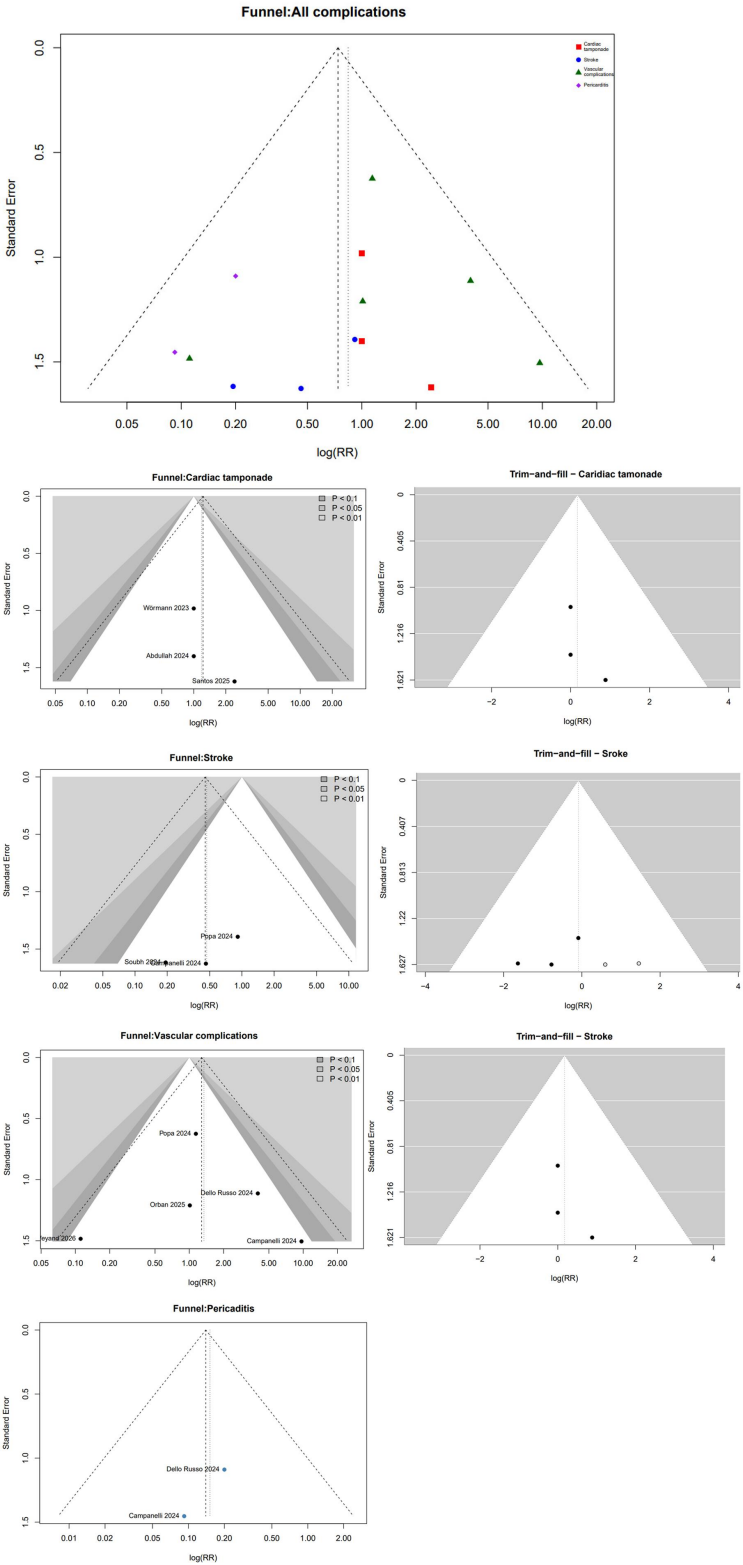
Funnel plot.

For each complication classification subgroup, due to the small number of included studies in a single subgroup, the statistical power of the conventional publication bias test was insufficient. The evaluation results of each subgroup were as follows: the funnel plot and the Duval and Tweedie's trim-and-fill method correction plot of each complication classification showed that no obvious publication bias was detected, and only 2 potentially missing studies were estimated for the stroke subgroup, and the core conclusions were not changed after correction. Pericarditis was a complication that could not be tested statistically because of the small number of included studies, but the funnel plots showed symmetry (Figure 16).

#### 3.4.3 Surgical characteristics

##### 3.4.3.1 Procedure time

###### 3.4.3.1.1 Main analysis results

**Figure 17:**
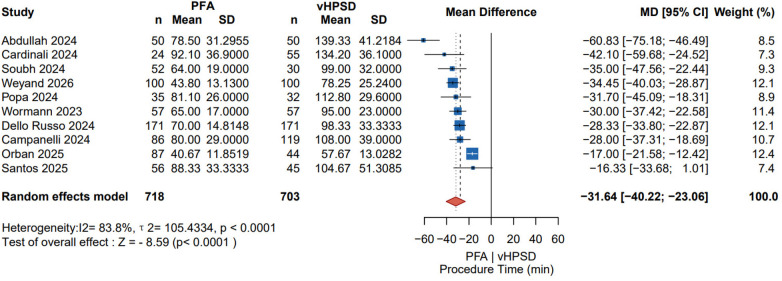
Forest plot of the main analysis results.

This study further analyzed the procedure time of the two ablation techniques. There were 10 studies reporting this outcome measure, with a total of 1,421 patients, including 718 in the PFA group and 703 in the vHPSD group. The pooled results showed that: The procedure time was significantly shorter in the PFA group than in the vHPSD group, with a pooled Mean Difference (*MD*) of −31.64 min (95%CI: −40.22 to −23.06, *P* < 0.0001, Figure 17), suggesting that PFA technique could shorten the procedure time by about 31.6 min on average, and significantly improve the efficiency of the operation. There was a high degree of heterogeneity among studies (*I^2^* = 83.8%, *P* < 0.0001), suggesting that there was a dispersion in the difference in procedure time between different studies. Of note, despite the high heterogeneity, the direction of effect was exactly the same in all the included studies, suggesting that the procedure time in the PFA group was shorter than that in the vHPSD group, with differences only in the magnitude of reduction. This may be related to differences in operator experience, standardization of surgical procedures, and baseline characteristics of patients across different centers.

###### 3.4.3.1.2 Subgroup analysis

**Figure 18:**
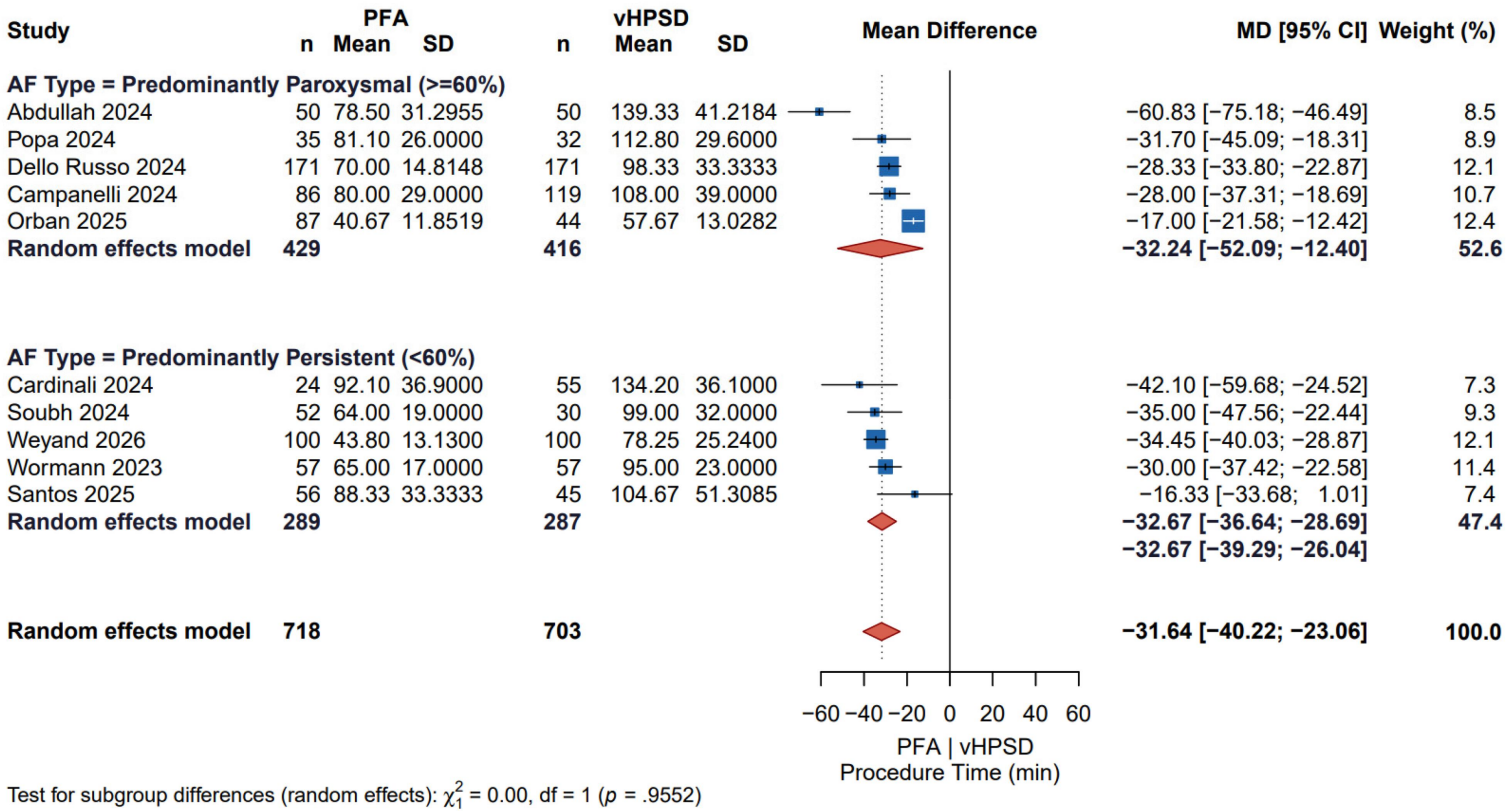
Forest plot of the subgroup analysis.

Subgroup analysis showed that the procedure time in the PFA group was significantly shorter than that in the vHPSD group, regardless of the type of AF, and the magnitude of the reduction was highly consistent: Subgroup dominated by paroxysmal AF: Five studies involving 845 patients (429 in the PFA group and 416 in the vHPSD group, Figure 18) were included. The combined effect size showed that the average procedure time was 32.24 min shorter in the PFA group (95%CI: −52.09 to −12.40, *P* < 0.001). Subgroup dominated by persistent AF: Five studies involving 576 patients (289 in the PFA group and 287 in the vHPSD group) were included. The combined effect size showed that the average procedure time was 32.67 min shorter in the PFA group (95%CI: −39.29 to −26.04, *P* < 0.001).

The results of subgroup difference test showed that AF type did not significantly modulate the procedure time difference between the two ablation techniques (*χ^2^*_1_ = 0.00, *df* = 1, *P* = 0.9552), suggesting that the conclusion of this study that PFA technique can significantly shorten procedure time has good consistency and extrapolation in patients with different AF types.

###### 3.4.3.1.3 Sources of heterogeneity (meta-regression)

To explore potential sources of heterogeneity among the included studies, univariate meta-regression analysis was performed to assess five prespecified baseline clinical covariates: proportion of paroxysmal AF, mean age of the study population, proportion of males, left ventricular ejection fraction (LVEF), and CHA_2_DS_2_-VASc score.

The results showed that none of the included covariates were statistically associated with the study effect size, and the exponential transformation value of regression coefficients suggested that there was only a small non-significant fluctuation in the risk ratio of AF-free rate during follow-up for each 1-unit increase in each covariate.

**Figure 19:**
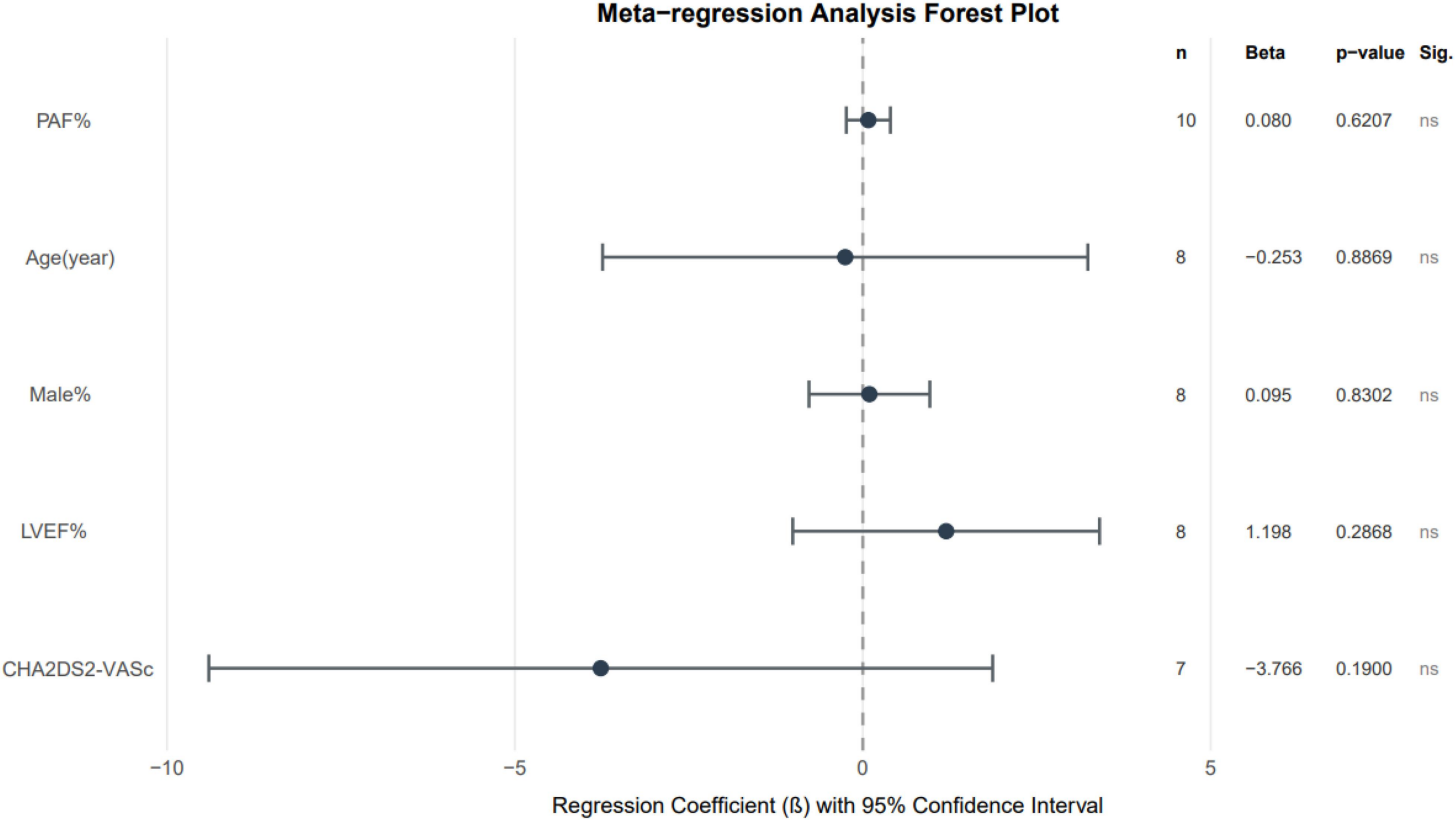
Meta-regression.

This finding suggests that the observed heterogeneity across the included studies was not due to differences in these common baseline clinical characteristics (Figure 19).

###### 3.4.3.1.4 Sensitivity analysis

Sensitivity analyses confirmed the robustness of the procedure-time result. The pooled *MD* remained consistently negative across alternative pooling models, exclusion of conference abstracts, and leave-one-out analyses, with no reversal of statistical significance. Reanalysis using *SMD* instead of *MD* also supported a significantly shorter procedure time in the PFA group. These findings indicate that the observed reduction in procedure time was not materially affected by model selection, study type, effect measure, or any single study. (Supplementary Table S2 and Supplementary Figures S3–S5).

###### 3.4.3.1.5 Publication bias

**Figure 20:**
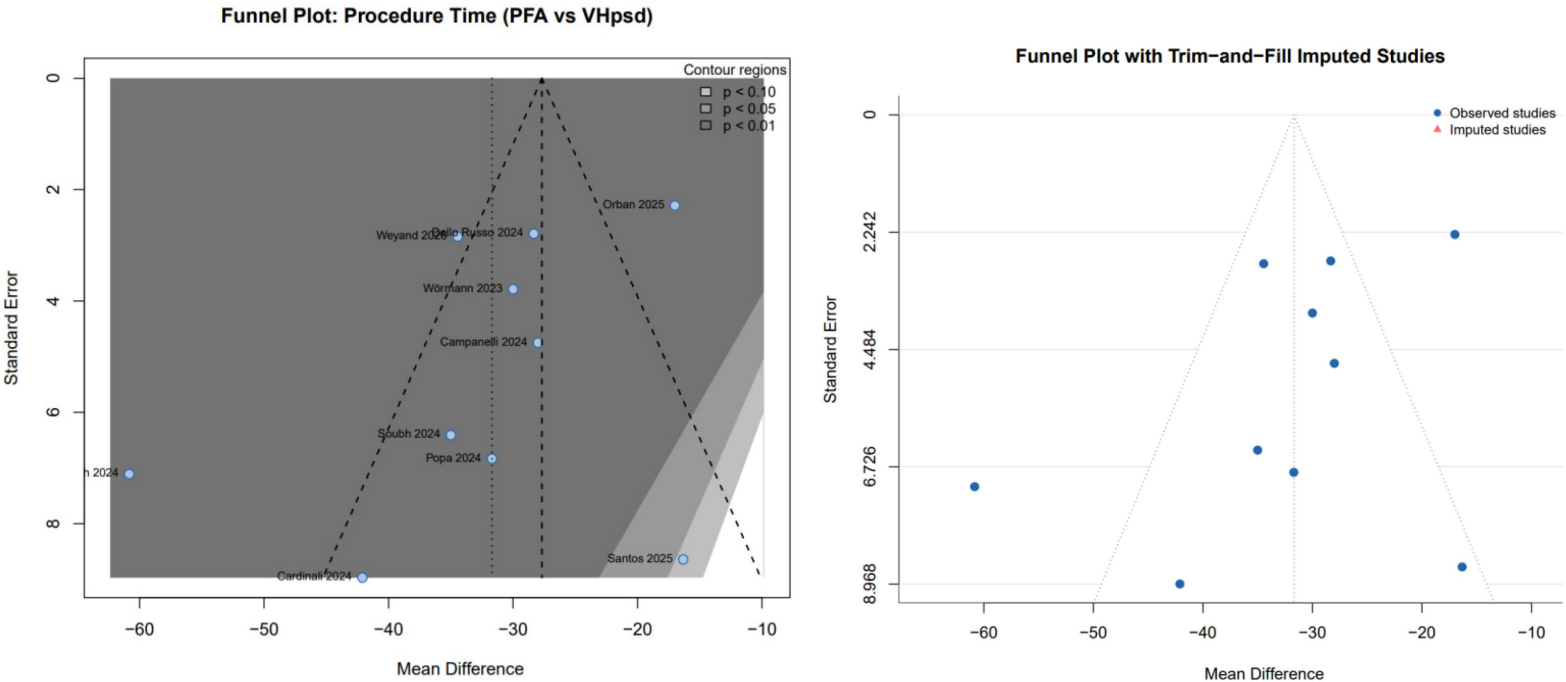
Funnel plot.

The funnel plot showed a generally symmetrical distribution with no obvious asymmetry. Begg's test yielded *P* = 0.3252 and Egger's test yielded *P* = 0.1345, with neither test detecting significant publication bias. Further application of the Duval and Tweedie trim-and-fill method did not estimate any potentially missing studies in the funnel plot, and the pooled MD remained −31.64 (95%CI: −40.22 to −23.04, *P* < 0.0001) both before and after imputation, suggesting that the procedure time was significantly shorter in the PFA group than in the vHPSD group. Overall, no publication bias was detected in this study, and bias correction caused no change in the pooled effect estimate or the direction of effect, confirming that the core conclusion remains of good reliability (Figure 20).

##### 3.4.3.2 Fluoroscopy time

###### 3.4.3.2.1 Main analysis results

**Figure 21:**
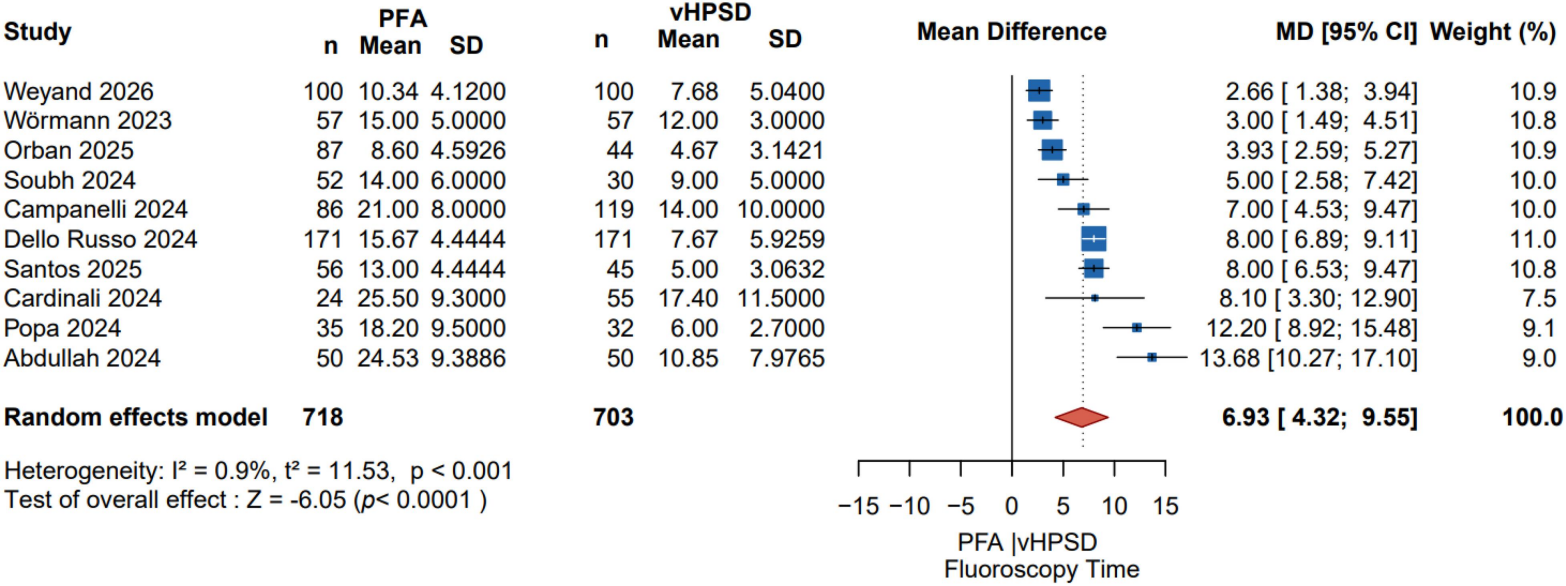
Forest plot of the main analysis results.

This study further analyzed the fluoroscopy time of the two ablation techniques. There were 10 studies reporting this outcome measure, with a total of 1,421 patients, including 718 in the PFA group and 703 in the vHPSD group. The pooled results showed that: Fluoroscopy time was significantly longer in the PFA group than in the vHPSD group, with a pooled Mean Difference (*MD*) of 6.93 min (95%CI: 4.32 to 9.55, *P* < 0.0001, Figure 21), suggesting that the average fluoroscopy time of PFA technique increased by about 6.9 min, which may increase the potential risk of radiation exposure to some extent. There was very low heterogeneity among studies (*I^2^* = 0.9%, *P* < 0.001), suggesting that the results were highly consistent across studies, and no significant dispersion was observed. It is worth noting that the direction of effect was completely consistent in all included studies, suggesting that the fluoroscopy time in the PFA group was longer than that in the vHPSD group, with only minor differences in the magnitude of the extension. This highly consistent result further verified the reliability of the conclusions of this study.

###### 3.4.3.2.2 Subgroup analysis

**Figure 22:**
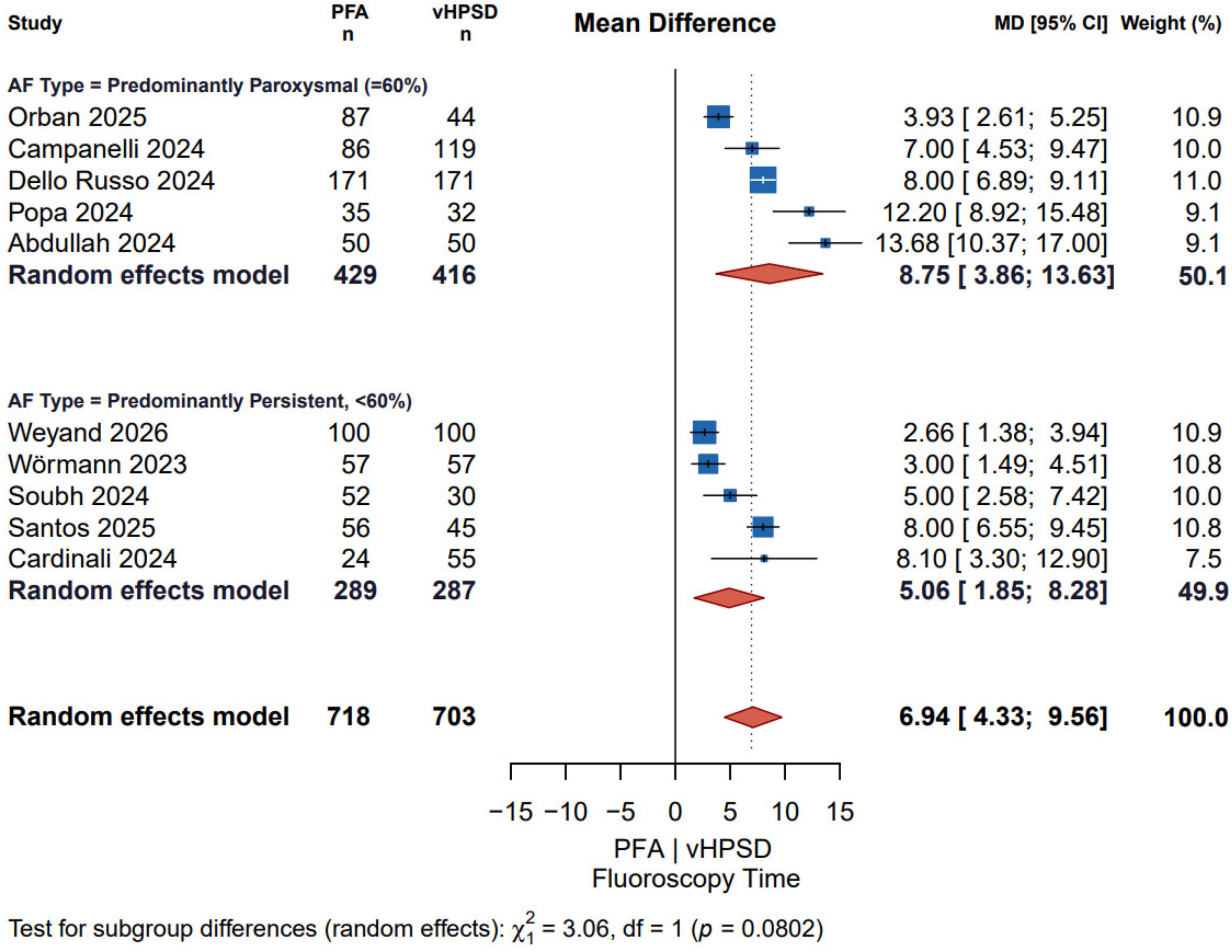
Forest plot of the subgroup analysis.

Subgroup analysis showed that fluoroscopy time was significantly longer in the PFA group than in the vHPSD group regardless of the type of atrial fibrillation, and the same direction of effect was observed in the two subgroups: in the subgroup dominated by paroxysmal atrial fibrillation: Five studies involving 845 patients (429 in the PFA group and 416 in the vHPSD group) were included (Figure 22). The combined effect size showed that fluoroscopy time in the PFA group increased by an average of 8.75 min (95%CI: 3.86 to 13.63, *P* < 0.001). Subgroup dominated by persistent AF: Five studies involving 576 patients (289 in the PFA group and 287 in the vHPSD group) were included. The combined effect size showed that fluoroscopy time in the PFA group increased by an average of 5.06 min (95%CI: 1.85 to 8.28, *P* < 0.01).

The results of subgroup difference test showed that AF type did not significantly adjust the difference of fluoroscopy time between the two ablation techniques (*χ^2^_1_* = 3.06, *df* = 1, *P* = 0.0802), suggesting that although the prolongation of fluoroscopy time with PFA technique in paroxysmal AF group was slightly higher than that in persistent AF group, the difference did not achieve statistical significance. The conclusion that the fluoroscopy time of the PFA technique is significantly longer than that of the vHPSD technique in this study has good consistency and extrapolation in people with different AF types.

###### 3.4.3.2.3 Sources of heterogeneity (meta-regression)

To explore potential sources of heterogeneity among the included studies, we performed univariate meta-regression analyses that assessed five prespecified baseline clinical covariates: proportion of paroxysmal atrial fibrillation (PAF), mean age of the study population, proportion of male patients, left ventricular ejection fraction (LVEF), and CHA2DS2-VASc score.

**Figure 23:**
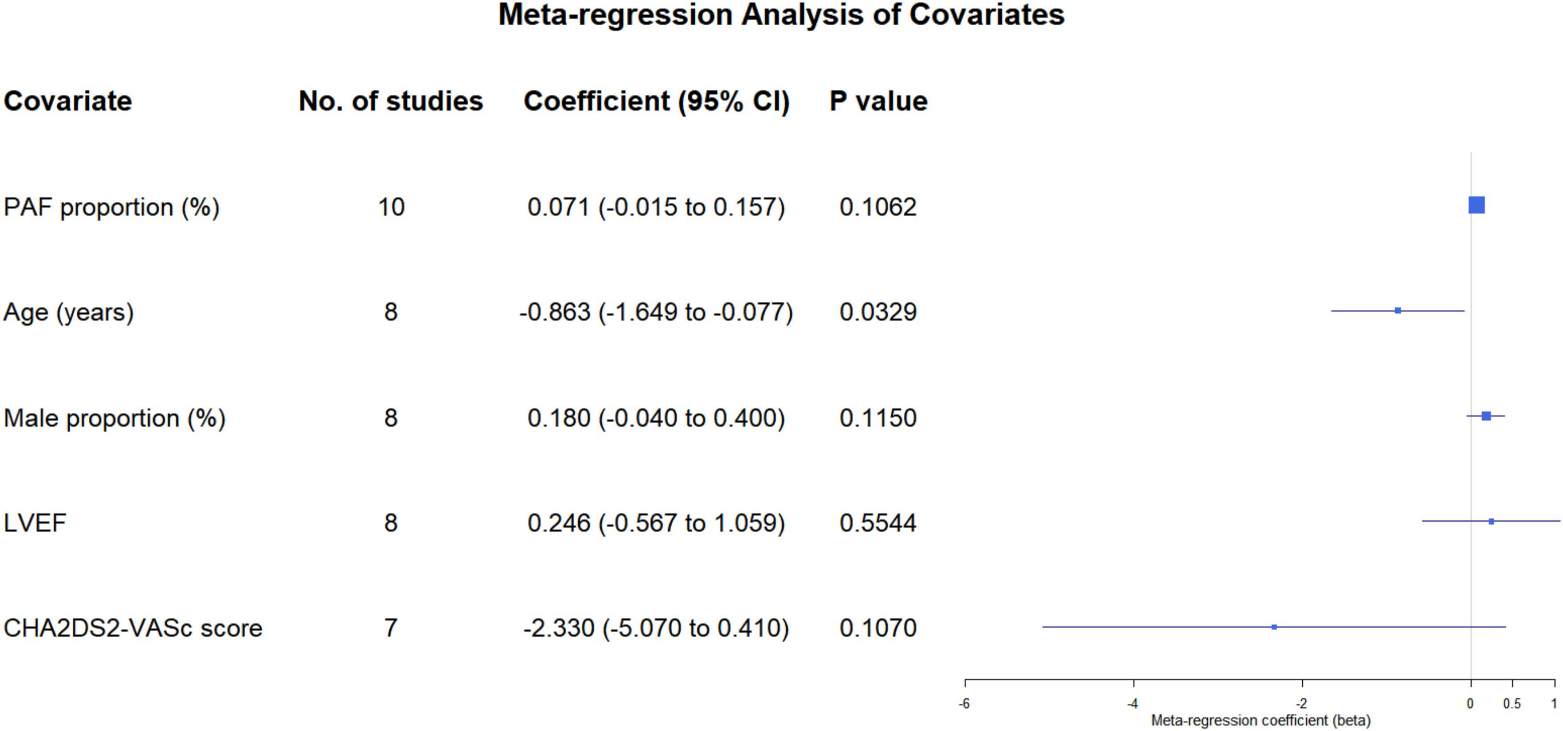
Meta-regression.

The results showed that only the effect size of the average age of the study population was statistically correlated with the fluoroscopy time (*β* = −0.875, *P* = 0.031), suggesting that the PFA-vHPSD decreased significantly with each increase of the average age of the study population. The remaining covariates included (proportion of PAF, proportion of males, LVEF) were not statistically associated with the study effect size (Figure 23).

###### 3.4.3.2.4 Sensitivity analysis

Sensitivity analyses supported the robustness of the primary finding for fluoroscopy time. Across alternative pooling models, the pooled *MD* ranged from 5.82 to 6.94 min, and all 95%CIs were above zero (Supplementary Table S2), consistently indicating a significantly longer fluoroscopy time in the PFA group than in the vHPSD group. After excluding studies published only as conference abstracts, the result remained significant and consistent with the main analysis (*MD* = 5.96 min, 95%CI: 2.87 to 9.06, *P* = 0.003, Supplementary Figure S3). Reanalysis using *SMD* instead of *MD* yielded the same conclusion (*SMD* = 1.13, 95%CI: 0.76 to 1.49, *P* < 0.0001, Supplementary Figure S4). Leave-one-out analysis showed that the pooled *MD* remained stable after sequential exclusion of individual studies, ranging from 6.19 to 7.42 min, suggesting that no single study materially influenced the pooled estimate (Supplementary Figure S5).

###### 3.4.3.2.5 Publication bias

**Figure 24:**
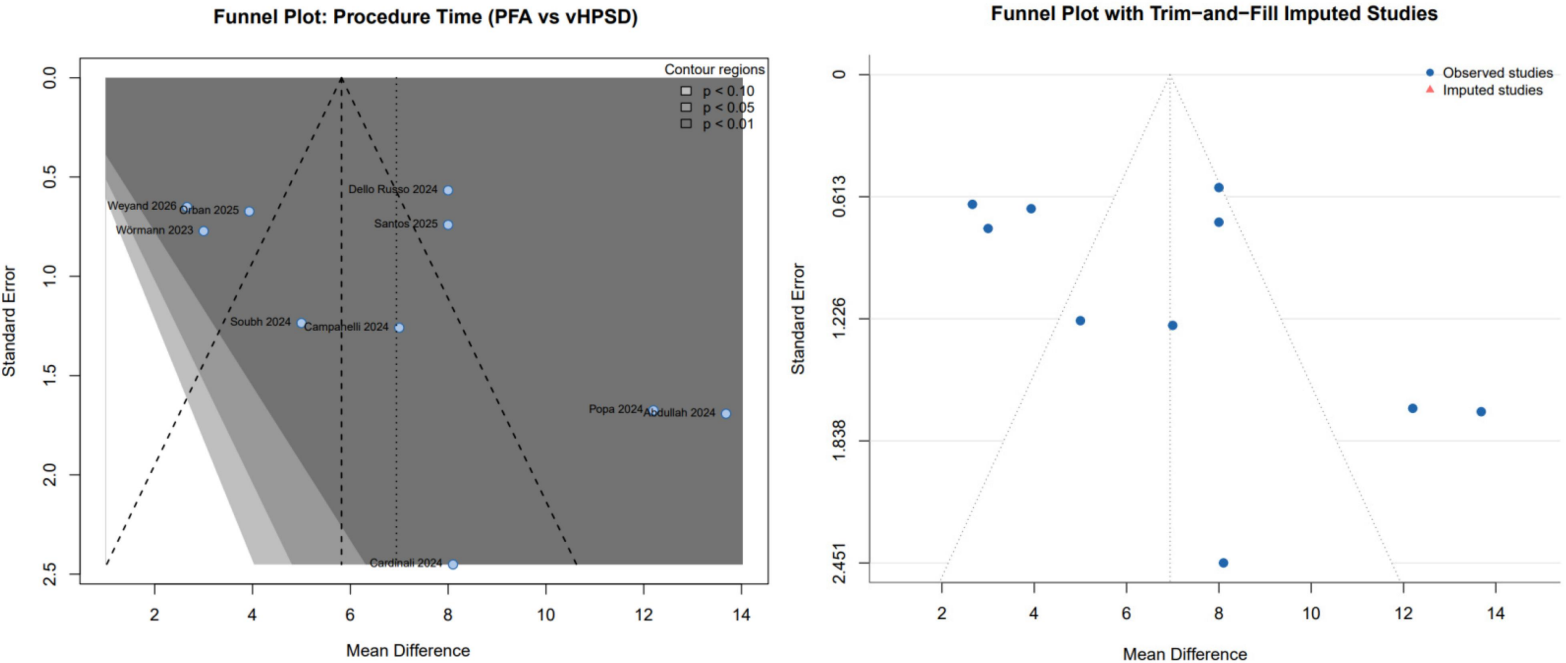
Funnel plot.

The funnel plot showed a generally symmetrical distribution with no obvious asymmetry. Begg's test yielded *P* = 0.2449 and Egger's test yielded *P* = 0.2491, with neither test detecting significant publication bias. Further application of the Duval and Tweedie trim-and-fill method did not estimate any potentially missing studies in the funnel plot, and the pooled *MD* remained 6.93 (95%CI: 4.33 to 9.56, *P* < 0.0001) both before and after imputation, suggesting that the fluoroscopy time was significantly longer in the PFA group than in the vHPSD group. Overall, no publication bias was detected in this study, and bias correction caused no change in the pooled effect estimate or the direction of effect, confirming that the core conclusion remains of good reliability (Figure 24).

##### 3.4.3.3 Fluoroscopy dose

Only 4 studies of fluoroscopy related radiation dose area product were reported. Due to the complex reporting units of the original literature, the great degree of dispersion of the values, and the high heterogeneity among the studies, quantitative meta-analysis was not performed and only descriptive statistical analysis was performed.

When converted to *μ*Gy m^2^, the radiation dose values of each group varied widely. The fluoroscopy radiation dose of PFA group tended to be higher than that of vHPSD group, but there was no unified quantitative evidence.

## 4 Discussion

A total of 11 observational studies (791 patients) were included in this study through the methodological framework of systematic search and meta-analysis. The aim of this study was to evaluate the efficacy (AF recurrence rate after the washout period, immediate pulmonary vein isolation rate), safety (overall complication rate and single complication rate), and procedural efficiency (total procedure time, fluoroscopy time and dose). A comprehensive and systematic comparative evaluation of PFA and vHPSD was performed. The overall analysis showed that there were no significant differences in AF recurrence rate, pulmonary vein isolation rate and complication rate between the two techniques. PFA has a significant advantage in procedure time, but it is accompanied by an increase in fluoroscopy time and dose. The following is an in-depth discussion of these core findings, including the mechanism basis, clinical connotation, consistency and divergence with the existing literature.

### 4.1 Comparability of baseline characteristics

In the meta-analysis of observational studies, the balance of baseline characteristics is the basic guarantee for the comparability between groups, and also the core prerequisite for reducing selection bias and maintaining the internal validity of the study. In this study, a pooled analysis of the baseline characteristics of the included literature was performed to assess the comparability of baseline characteristics among the studies. Overall, there were no significant differences in most baseline variables between the PFA group and the vHPSD group, including age, gender, CHA_2_DS_2_-VASc score, hypertension prevalence, left ventricular ejection fraction (LVEF) and other core clinical indicators, suggesting that the two groups were generally comparable. It provides a relatively reasonable baseline balance for subsequent comparison of efficacy and safety. Although there was moderate to high heterogeneity in some indicators, the combined effect size was still not statistically significant, which did not affect the conclusion of overall baseline balance, indicating that the differences between the studies may be due to regional differences and clinical differences, rather than systematic selection bias between the two groups. Of note, the variable age showed a statistical difference between the two groups (*MD* = −2.04, 95%CI −3.91 to −0.18), and although this small difference reached statistical significance, the mean age difference of 1.43 years is small from a clinical perspective and may have limited practical effect on AF ablation-related outcomes.

In addition, it is also noted in the present study that patients with repeat ablation who had already undergone failed pulmonary vein isolation were also included in the studies by Dello et al. and Orban et al. Previous ablation history itself may affect the risk of recurrence, the pattern of pulmonary vein reconnection, the difficulty of repeat ablation, and the risk of complications. Due to the lack of access to the individual original data of the study, we could not conduct a separate exclusion analysis for these patients, and this factor may theoretically bring a certain risk of bias. Therefore, this study can only conclude that the PFA group and the vHPSD group are generally comparable in terms of extractable baseline variables and cannot further infer that the two groups are fully balanced in all key factors affecting prognosis.

In summary, the available evidence generally supports that the PFA group and the vHPSD group are basically comparable in baseline characteristics, and the comparison between groups in this meta-analysis is based on a relatively reasonable baseline balance. However, given the nonrandomized observational design of all the included studies, the potential influence of residual confounding variables (such as operator experience, center surgical volume, patient compliance, and antiarrhythmic drug use strategy) on the final efficacy and safety conclusions cannot be completely excluded. Future high-quality randomized controlled trials will be helpful to further verify the robustness of the conclusions of this study.

### 4.2 Equivalence of efficacy

On the core efficacy endpoint of AF treatment, our results show that two ablation techniques based on completely different mechanisms of action ultimately achieve highly similar clinical efficacy.

#### 4.2.1 Recurrence rate of atrial fibrillation

In terms of AF recurrence, our meta-analysis suggested that there was no significant difference between PFA and vHPSD in the incidence of AF recurrence after blanked-period (*RR* 0.99, 95%CI 0.91 to 1.07, *P* = 0.722). A number of meta-analyses have compared PFA with radiofrequency ablation, but most of them use “HPSD” as a unified classification and fail to distinguish between HPSD(45–50W) and vHPSD(>70W), which are fundamentally different in energy parameters, lesion characteristics, and clinical outcomes. For example, Amin et al. showed that PFA significantly reduced atrial arrhythmia recurrence compared with standard HPSD (45–50W) (*RR* 0.69, 95%CI 0.54 to 0.88, *P* < 0.01) in a meta-analysis of 7 observational studies involving 1,904 patients. However, compared with vHPSD (70–90W), there was no significant difference in recurrence rate between the two groups (22). Traditional radiofrequency ablation requires point by point for a long time, which is prone to problems of insufficient transmural properties and uneven damage, while vHPSD produces transmural myocardial necrosis in a very short time through extremely high power (5, 6), The transmural permeability and persistence of the lesions were significantly improved, thus narrowing the gap between the long-term efficacy and PFA. PFA has the advantage of irreversible electroporation mechanism and tissue selectivity, which can effectively ablate myocardial tissue while retaining the extracellular matrix (8, 9). It is against this background that this study focused on the direct comparison of PFA and vHPSD, filling the gap of the existing meta-analysis in the comparison of technical stratification, and providing an evidence-based basis for the selection of PFA and vHPSD for clinical practice.

Another meta-analysis focused on vHPSD (70–90W) similarly confirmed that PFA and vHPSD were equally effective in maintaining sinus rhythm after surgery (*RR* 1.03, *P* = 0.5) (23). It is worth noting that although there is no statistical difference, it still needs to be interpreted in clinical practice according to specific patient characteristics: Mariani et al. performed a subgroup analysis of 1,190 patients by AF type, and the results showed that the incidence of paroxysmal AF (*OR* 0.74, 95%CI 0.50 to 1.11, *P* = 0.14) and persistent AF (*OR* 0.68, 95%CI 0.35 to 1.34, *P* = 0.14) was significantly higher than that of paroxysmal AF (*OR* 0.74, 95%CI 0.50 to 1.11, *P* = 0.27) (24), Given the wide confidence intervals in the subgroup analyses described above, the substantial significance of this trend needs to be confirmed by studies with larger samples.

In addition, there was considerable heterogeneity in the duration of follow-up in the included literature, which ranged from 3 months to 12 months, and this heterogeneity itself leads to potential interference in the interpretation of the results. To systematically evaluate the moderating effect of different follow-up duration on the pooled effect size, we included follow-up duration as a covariate in the meta-regression analysis, and no significant moderating effect was found. However, this negative result needs to be interpreted cautiously. Due to the small number of studies and the small number of patients included in this study, the statistical power of the meta-regression analysis itself is insufficient, and the possibility of follow-up time as an effect modifier cannot be reliably excluded. This negative result cannot be equated with a confident judgment that follow-up time has no effect on the results.

The duration of follow-up in this study was short to medium term, which fundamentally limits the ability of our conclusions to be extrapolated to long-term treatment effects. Long-term follow-up data of traditional radiofrequency ablation have consistently shown that the recurrence rate of atrial fibrillation after catheter ablation shows a continuous increasing trend with the extension of follow-up time (25). This rule suggests that the conclusion of “efficacy equivalence” based on the results of short—and medium-term follow-up may not reflect the true difference in efficacy between the two techniques in the longer term. From the injury perspective, the chronic remodeling process of the ablation site, including the maturation and contraction of the fibrotic scar, the functional recovery of the remaining cardiomyocytes, and the slow formation of conduction gaps in the distal pulmonary veins, usually continues from 6 months to 2 years after the ablation procedure. The question is whether the essential difference in lesion morphology between PFA and vHPSD can be translated into a detectable difference in long-term pulmonary vein reconnection rate (26, 27), There is currently no direct clinical evidence to answer.

At present, there is a lack of high-quality evidence to directly compare the long-term efficacy of PFA and vHPSD ablation over 2 years, which constitutes an important gap in the current evidence system that cannot be ignored. In the future, there is an urgent need to design prospective long-term follow-up studies specifically for the above two techniques to fully reveal the potential differences in long-term sinus rhythm maintenance between the two techniques, and to provide a more comprehensive and reliable evidence-based basis for clinical technology selection.

#### 4.2.2 Immediate pulmonary vein isolation rate

The immediate success rate of pulmonary vein ablation is the key process indicator to reflect the efficacy of the ablation technique in the acute phase. The present study showed that the immediate pulmonary vein isolation rate was almost 100% in both PFA and vHPSD, and there was no statistical difference between the two groups. This is consistent with the results of the meta-analysis published by Iqbal et al., which included four observational studies with 605 patients and showed that the PVI success rate of PFA and vHPSD was 100% (*RR* 1.00, 95%CI 0.99 to 1.01, *P* = 1) (23). It is concluded that under the current mature device platform and standardized operation process, the advantages and disadvantages of the two techniques cannot be distinguished simply by the immediate pulmonary vein isolation rate, and the focus of clinical evaluation must shift to the durability of injury.

In this context, data on the rate of pulmonary-vein reconnection are particularly critical, but this is an important limitation of our study. The included literature did not provide detailed data on rates of pulmonary-vein reconnection, preventing a direct comparison of the two techniques on this key end point. The current indirect evidence comes from the meta-analysis conducted by Pranata et al., who reconstructed individual patient data and found that the rate of pulmonary vein reconnection in the PFA group and the HPSD/vHPSD group was 32% and 35%, respectively (*OR* 0.84, *P* = 0.473) (28), This suggests that there may be no significant difference between the two groups in the durable isolation of pulmonary veins. However, this study failed to strictly distinguish between standard HPSD and vHPSD, and its reference value for vHPSD is limited. This illustrates the urgent need to conduct long-term follow-up studies specifically targeting PFA and vHPSD to systematically collect data on the rate of pulmonary vein reconnection, which is one of the most important research gaps revealed by this meta-analysis.

### 4.3 Overall safety profile

#### 4.3.1 Overall complications

In terms of overall complications, both PFA and vHPSD showed high safety. The pooled analysis of this study showed that there was no significant difference in the overall incidence of complications between the two groups (*RR* = 0.89, 95%CI: 0.58 to 1.35, *P* = 0.5353). Consistent with the findings reported by Mariani et al., there was no significant difference in total complications (*OR* 0.92, *P* = 0.81) (24), This finding suggests that both techniques have acceptable perioperative safety under the current study conditions.

However, the overall equivalent conclusion masks the inherent structural differences in the complication spectrum, and if safety is measured only by overall complications, it will cause serious misjudgment of the two techniques. In a network meta-analysis, the overall complication rate was higher with PFA (29), This discrepancy may be due to differences in the definition of complications, with this analysis including a wide range of events, including phrenic nerve injury and esophageal injury, whereas other studies may have included only severe complications. This suggests that a standardized definition and grading system for complications is critical in the interpretation of complication data, and it is also a methodological issue that must be addressed in future study designs.

In view of the fundamental differences between PFA and vHPSD in terms of damage mechanism and energy delivery mode, different ablation methods have their own specific complications spectrum, so it is necessary to analyze the sub-components of various complications. Separate pooled analyses were performed for cardiac tamponade, stroke, vascular complications, and pericarditis.

#### 4.3.2 Single complication

##### 4.3.2.1 Cardiac tamponade

Three studies (315 patients) were included in this study. The pooled results showed that there was no significant difference in the incidence of cardiac tamponade between PFA group and vHPSD group (*RR* = 1.19, 95%CI 0.29 to 4.88, *P* = 0.809).

In terms of mechanism, the main driver of cardiac tamponade in catheter ablation is myocardial perforation caused by transatrial septal puncture and intracardiac ablation catheter manipulation, rather than the thermodynamic effect of ablation energy itself (30). No matter which ablation method is used, transseptal puncture is an inevitable procedure. The risk of atrial septal puncture largely depends on the experience of the operator and anatomical variation, rather than the ablation method itself. The pooled results of this study are consistent with the meta-analysis by Mariani et al. who reported no significant difference in the incidence of cardiac tamponade between the PFA and RFA groups (*P* = 0.80) (24), further supporting the essential equivalence of the two techniques in the risk of cardiac perforation.

However, the statistical limitations must be considered when interpreting this result. Only 3 studies included in this study reported this index, the sample size was only 315 cases, and the confidence interval of the combined effect size was extremely wide, which led to the serious lack of power of the combined results. Therefore, the conclusion that there was no significant difference in the incidence of cardiac tamponade should be interpreted as an inability to detect a difference in the incidence of cardiac tamponade in the literature included in this study, rather than an equivalence. This difference suggests that future studies with a larger sample size are needed to adequately compare this deadly complication.

##### 4.3.2.2 Stroke

Three studies (346 patients) were included in this study, and the pooled results showed that there was no significant difference in the incidence of perioperative stroke between the two groups (*RR* = 0.47, 95%CI 0.08 to 2.66, *P* = 0.392). Although the difference was not statistically significant, it was still worthy of further analysis.

From the perspective of energy-blood interaction, radiofrequency ablation (RFA), as a thermal ablation, will produce inevitable local high temperature at the catheter-blood interface, trigger blood protein denaturation and coagulation activation, form carbonization and microthrombus, and bring the risk of perioperative embolism (31).Although vHPSD greatly reduces the accumulation of thermal effect by shortening the ablation time. However, the instantaneous high temperature generated by resistive heating still has the possibility of contact with blood. In contrast, PFA, as a non-thermal electroporation technique, energy delivery is independent of temperature, eliminating the pathological basis of heat-induced coagulation from the root, without catheter carbonization and thrombosis (32), PFA does not appear to lead to thrombosis. Although PFA avoids the risk of thermal thrombosis caused by thermal ablation, its own non-thermal damage also brings new potential thrombotic risks. Zhou et al. reported four potential mechanisms for delayed cerebrovascular events after PFA: endothelial cell injury releasing procoagulant microparticles, endothelial dysfunction, blood stasis in large caliber sheath tubes, and an indirect procoagulant effect of PFA-related hemolysis (33). Therefore, the “potential advantage” of PFA in cerebrovascular events remains uninferred at present and lacks the support of a large sample.

From a statistical perspective, the confidence intervals in this study are extremely wide, and since stroke itself is a rare complication of catheter ablation, the underlying incidence is only 0.1% to 0.5% (34), This means that even if a large number of studies were pooled, it would be extremely difficult to achieve sufficient power to detect subtle differences between the two techniques. Therefore, under the current evidence-based evidence, we cannot hide the potential advantages of PFA by stating that there is no statistical difference between the two techniques.

##### 4.3.2.3 Vascular complications

A total of 5 studies (945 patients) were included in this study, and the pooled results showed that there was no statistically significant difference between the two groups (*RR* = 1.35, 95%CI 0.56–3.25, *P* = 0.508).

The delivery sheath of PFA (represented by FARAPULSE™) (13–15Fr) is much larger than that of the standard radiofrequency sheath (8.0–8.5fR). Theoretically, it will increase the risk of complications such as hematoma and pseudoaneurysm at the puncture site (35). However, the pooled analysis of this study showed that there was no significant difference in vascular access complications between PFA and vHPSD, only the point estimate of PFA group was slightly higher (*RR* = 1.35). We tentatively interpret that the risk of large sheath can be effectively controlled by mature techniques such as ultrasound-guided puncture, lengthening compression, and vascular stapler. In addition, PFA has shorter procedure time and sheath indwelling time, which offsets part of the trauma. At the same time, different definitions of complications in different studies and insufficient power due to low event rates may also affect the results.

Although the difference did not reach statistical significance, we should also pay attention to this numerical trend (*RR* = 1.35), reminding surgeons that they should pay extra attention to vascular access management when using PFA system. Preoperative vascular access conditions should be fully evaluated and corresponding preventive measures should be taken.

##### 4.3.2.4 Pericarditis

The most clinically important and academically significant finding of this study was the significant difference in the incidence of pericarditis: the pooled analysis based on 2 studies and 547 patients showed that the incidence of pericarditis was significantly lower in the PFA group than in the vHPSD group (*RR* = 0.15, 95%CI 0.03 to 0.84, *P* = 0.030).

vHPSD is a thermal ablation, which causes coagulation necrosis through the pyroptosis, cell membrane rupture, massive release of DAMPs, activation of TLR/NLRP3 pathway, and induction of intense inflammation, which can involve the epicardial and pericardium, leading to sterile pericarditis 24–72 h after surgery, and even post-ablation syndrome (36). PFA is a non-thermal ablation, which causes a mixture of apoptosis and electroporation necrosis by irreversible electroporation. Apoptosis is “immune silence”, without the release of a large number of DAMPs, and the inflammatory activation is weak (33). Therefore, PFA has a clear mechanism advantage in reducing perioperative pericarditis.

The results of the pericarditis subgroup analysis in this study are not isolated findings; Amin et al. also reported that PFA was associated with a significantly reduced risk of pericarditis, with a statistical advantage over both HPSD (*P* = 0.003) and vHPSD (*P* = 0.019) (22). The study by Malik et al. clearly stated that the incidence of cardiac tamponade was lower in the PFA group than in the HPSD group (37).This is consistent with the conclusion of this study.

Although this finding is statistically significant and clearly supported by theory, caution is needed about the robustness of the conclusions. First, the analysis was supported by only two studies, which is still a limited sample and has wide confidence intervals. Secondly, the two included studies may differ in the diagnostic criteria and severity of pericarditis. We look forward to including more high-quality studies for verification in the future.

#### 4.3.3 Specific complications of PFA

One of the studies included in this study reported transient intravascular hemolysis events after surgery (19). The hemolytic manifestations documented in this report were transient and resolved spontaneously without serious clinical consequences (38), this signal is not an occasional isolated phenomenon—it fits well with reports of adverse effects of PFA that have continued to accumulate in larger clinical practice in recent years. It suggests that it is necessary for us to examine the problems reflected behind the signal beyond the superficial description.

With the increase of clinical use, the complications spectrum of PFA has become clearer. Although it reduces some traditional thermal injury, it brings new risks, among which the most noteworthy complications are intravascular hemolysis and acute kidney injury. PFA induces the increase of erythrocyte membrane permeability and even rupture by high pressure pulse, leading to the release of free hemoglobin into the blood, which leads to hemolysis (39). Recently, Kuraoka et al. showed that plasma free hemoglobin, LDH, and total bilirubin levels were significantly increased and haptoglobin was significantly decreased after PFA compared with traditional radiofrequency ablation, suggesting that PFA does cause different degrees of subclinical hemolysis (40). The study by Barbosa et al. similarly showed that PFA was associated with more pronounced perioperative hemolysis and a higher risk of acute kidney injury (AKI) compared with RFA (41). The study by Nies et al. confirmed that PFA could induce dose-dependent hemolysis, and the degree of hemolysis increased with the increase of the frequency of discharge, and the hemolysis was more obvious when the catheter had poor contact with the tissue, which was especially obvious in patients with persistent atrial fibrillation (42). The complex substrate modification needs of persistent AF may lead to more discharges, so the risk of renal injury in persistent AF is also greatly increased.

In summary, the complication spectrum of PFA should be understood as risk transfer rather than risk disappearance, and we should pay attention to the complication of hemolysis, which can be effectively avoided through standardized operation and management.

### 4.4 Surgical characteristics

Surgical efficiency is one of the important dimensions to evaluate the clinical applicability of ablation techniques. The surgical efficiency index in this study covers the total procedure time, fluoroscopy time and radiation exposure dose. In this study, a significant and consistent advantage of PFA was observed in terms of procedure time, which could reduce the total procedure time by an average of about 31.6 min. However, PFA showed an opposite result in terms of fluoroscopy time, which was about 6.9 min longer than vHPSD on average. However, this advantage is accompanied by an increase in fluoroscopy time and dose.

#### 4.4.1 Procedure time

The pooled analysis of 10 studies involving 1,421 patients showed that the total operative time in the PFA group was significantly shorter than that in the vHPSD group (*MD* = −31.64 min, 95%CI: −40.22 to 23.06, *P* < 0.0001). Consistent with the results of network meta-analysis by Deepan et al., the procedure time of PFA ranked first (*P*-score: 100%) (29). This time advantage is not only highly significant statistically, but also has a non-negligible significance in clinical practice. Although there was a high heterogeneity among the studies (*I^2^* = 83.8%), all the included studies remained completely consistent in the direction of effect, which pointed to the core conclusion of “shorter PFA procedure time”, and the difference was only in the magnitude of reduction.

From the perspective of mechanism, the advantage of PFA procedure time is not caused by a single factor, but is driven by the following factors. First, the five-valve flowershaped catheter design of PFA can be used to complete a 360° circumferential pulmonary vein ablation in a single unfolding, which subverted the traditional point-by point sequential ablation paradigm. Although vHPSD reduces the ablation time of a single point, it still needs 15 to 25 ablation points to complete single pulmonary vein isolation. The efficiency of VHPSD is limited by the topology of point-by-point accumulation (43). Secondly, PFA uses a millisecond pulsed electric field, and the energy-effect conversion is completed immediately without waiting for thermal storage. The overall ablation time of PFA is much lower than that of vHPSD mode (44). Third, PFA simplifies the safety monitoring process and further shortens the procedure time by eliminating the need for routine placement of esophageal thermometry probes to avoid thermal damage due to myocardial selectivity (45).

The interpretation of high heterogeneity mainly includes the following aspects: There are obvious technical differences between the PFA operation paradigm and vHPSD. The PFA procedure time of the centers in the early stage of the learning curve is significantly longer than that of the experienced centers. The procedure time decreased steadily from 77.9 min to 62.3 min, with a decrease of 20.1%. The study also demonstrated a 45.9% decrease in fluoroscopy time, and the proportion of zero-fluoroscopy procedures could be optimized simultaneously (46); Second, the different ablation strategies, some centers performed additional ablation sites after the completion of standard pulmonary vein isolation, which directly prolonged the total procedure time. Persistent atrial fibrillation usually requires longer procedure time due to complex mechanism modification. The difference in the proportion of the two types of atrial fibrillation in the studies included in this study constitutes an important source of heterogeneity.

#### 4.4.2 Fluoroscopy time

The present study, also based on a pooled analysis of 1,421 patients from 10 studies, found that while the total time of PFA procedure was significantly shorter, the fluoroscopy time was significantly longer than that of vHPSD (*MD* = 6.93 min, 95%CI: 4.32 to 9.55, *P* < 0.0001), and there was little heterogeneity (*I^2^* = 0.9%) of this opposite finding across the included studies. The highly consistent direction of effect indicates that this phenomenon is not a random error due to chance, but rather a systematic rule determined by the intrinsic characteristics of the two techniques.

The core reasons for the shorter fluoroscopy time of vHPSD than PFA are as follows: The first is the fluoroscopy dependence of the instrument itself. PFA requires multi-position fluoroscopy to confirm the integrity of the petal of the catheter and to exclude the risk of damage of the catheter embedded in the pulmonary vein. Secondly, vHPSD relies on the mature RF platform and is highly integrated with the 3D mapping system, while PFA still has limitations in integration with the mapping system. However, the new generation of PFA has been promoting deep integration with CARTO and other platforms, and this disadvantage is expected to be reduced in the future (47). In addition, as mentioned above, the learning curve is different due to the different surgical experience of the surgeons, which leads to the need for fluoroscopy time to compensate.

It is worth pointing out that “zero perspective” or “near zero perspective” PFA procedures have made positive progress in clinical practice. A number of studies have confirmed that PFA can safely complete pulmonary vein isolation under very low or near zero fluoroscopy, using intracardiac ultrasound (ICE) to guide atrial septal puncture and catheter localization in real time, combined with three-dimensional mapping system to assist tracking, and the surgical effect is not significantly different from that of conventional fluoroscopic guidance (48, 49). With the standardization and promotion of zero-perspective technique and the deepening integration of PFA and three-dimensional mapping platform, the current disadvantage of fluoroscopy time of PFA is expected to be fundamentally reversed in future technical iterations, and finally realize the double optimization of procedure time and fluoroscopy time.

#### 4.4.3 Fluoroscopy dose

In this study, only 4 studies reported the fluoroscopy radiation dose data, and there was obvious methodological heterogeneity in the reporting institutions of each study, which led to the fundamental limitation of the technical feasibility of quantitative meta-analysis. Only descriptive analysis was performed on this index in this study. Nevertheless, the descriptive data converted to μGy·m^2^ showed a directional signal worth attention at the level of numerical trend: the fluoroscopy radiation dose in the PFA group tended to be higher than that in the vHPSD group in most reported studies.

We recommend that future prospective studies comparing PFA and vHPSD include radiation dose as a prespecified standardized secondary endpoint, and unify the following key methodological elements in study design: ① Unify reporting metrics—use the dose-area product (DAP, Gy·cm^2^) as the standard radiation dose reporting unit; ② Control of equipment factors: in the design stage, the types of fluoroscopy equipment and dose rate patterns of participating centers were pre-registered and stratified. ③ Unified statistical range—clearly defined operation stages included in radiation dose statistics. Only when the above methodology is systematically implemented, future meta-analysis will be able to achieve a meaningful quantitative combination of radiation doses, so as to provide truly reliable quantitative evidence for the comparison of the two techniques in the radiation safety dimension.

### 4.5 Persistent AF: a differentiated advantage that has not yet been defined

The results of this study mainly reflect the overall effect of PFA and vHPSD in mixed AF population. Limited by the data reporting form of the included studies, it is unable to directly answer whether there are differences in efficacy between PFA and VHPSD in different AF subtypes. To overcome this limitation, the research team proactively contacted the corresponding authors of all the studies to obtain individual patient raw data stratified by AF type for subgroup analyses, but unfortunately, we were unable to obtain available raw data sets. This limitation is not only a methodological regret, but also an unavoidable background for understanding the applicability of our conclusions.

In the ablation of paroxysmal AF, pulmonary vein isolation is usually the core, and both PFA and vHPSD can achieve this goal more efficiently, so the conclusion that the efficacy of PFA and VHPSD is similar in this subtype has sufficient basis. However, the ablation strategy for persistent AF is much more complex than that for paroxysmal AF, and this complexity is the root cause of the different performance of the two techniques in persistent AF, which also makes the overall equivalence conclusion of this study should be used with caution in patients with persistent AF.

Persistent atrial fibrillation often requires not only simple pulmonary vein isolation, but also left atrial posterior wall isolation, mitral isthmus line, or other substrate modification due to its complex mechanism. However, the available evidence is still limited to support the use of PFA in such linear ablation. The MANIFEST-PF registry showed that PFA combined with left atrial posterior wall ablation did not further improve the 12-month atrial tachyarrhythmia free survival compared with PVI alone in patients with persistent AF (50). MANIFEST-REDO study showed that the risk of AT/AF recurrence after repeat ablation was significantly higher in patients with persistent AF as the type of recurrent arrhythmia (*HR* 1.241, 95%CI 1.534–1.005, *P* = 0.045) (51). The above evidence collectively suggests that the clinical benefit of PFA in modifying the complex matrix of persistent atrial fibrillation is not stable, and its augmentation effect remains to be clarified. In contrast, Compagnucci et al. showed that PVI+ left atrial posterior wall ablation (PWA) for vHPSD showed better feasibility and efficacy in patients with persistent atrial fibrillation (52). It is suggested that vHPSD may have more mature technical adaptability in the complex ablation scenario of persistent AF. Still, PFA is not without promise in patients with persistent AF, and Reddy et al. demonstrated that PFA is safe and effective for pulmonary vein isolation combined with left atrial posterior wall ablation in patients with persistent AF (53).

In conclusion, vHPSD currently appears to be the more mature and evidence-based technique option for patients with persistent AF who require combined linear ablation. However, the role of PFA in persistent atrial fibrillation needs to be clarified by long-term follow-up randomized controlled trials. Due to the lack of access to stratified raw data and subgroup analysis, the extrapolation of the equivalence conclusions of this meta-analysis in patients with persistent AF should be cautious. Future studies should prospectively stratified by AF type in the design stage to truly answer the core clinical question “whether the two techniques are equivalent in different AF patients”.

## 5 Limits

However, these efficacy and safety findings should be interpreted with caution. Several limitations may affect the robustness and generalizability of the pooled estimates: first, the included evidence was mainly derived from retrospective observational studies, prospective non-randomized controlled trials, and conference abstracts. Although we applied predefined inclusion and exclusion criteria, the non-randomized nature of most included studies means that selection bias could not be fully avoided. The limited number of studies and the relatively small overall sample size may also have reduced the statistical power of the analysis, especially for outcomes with low event rates.

The assessment of AF recurrence represents another important source of uncertainty. In clinical practice, the reported recurrence rate is closely related to the intensity and method of rhythm monitoring after ablation. The included studies mainly used Holter monitoring and ECG follow-up, while only some studies used cardiac implantable electronic devices. The studies also differed in their definitions of the blanking period, and several studies had relatively short follow-up durations. These differences may have led to an underestimation of the true recurrence burden and may have limited the comparability of recurrence outcomes across studies.

We originally planned to perform patient-level subgroup analyses to compare the effects of PFA and vHPSD separately in patients with paroxysmal AF and persistent AF. This approach would have provided more precise evidence for different clinical populations. We contacted the corresponding authors of all included studies to request raw patient-level data, but we did not receive responses and therefore could not obtain the necessary subgroup data. As an alternative, we performed study-level subgroup analyses by grouping studies according to whether they predominantly enrolled patients with paroxysmal AF, defined as at least 60% paroxysmal AF, or patients with persistent AF. This analysis allowed us to explore the possible influence of AF type on procedural time differences, but it could not replace a true patient-level analysis. Future large-scale studies should report more detailed stratified data so that subgroup effects can be assessed more accurately.

Some included studies did not directly report the mean and standard deviation for relevant outcomes. We therefore estimated these values from the reported median and interquartile range. This conversion method is commonly used in meta-analyses, but it relies on the assumption that the underlying data are approximately normally distributed. When the actual data distribution deviates from this assumption, the estimated values may introduce additional uncertainty into the pooled results. This issue should be considered when interpreting the magnitude and precision of the observed effects.

Baseline imbalance between comparison groups may also have affected the results. Our analysis of major baseline variables showed a statistically significant difference in age between groups. Younger patients may have a lower risk of arrhythmia recurrence regardless of the ablation strategy used. Because the number of included studies was limited, we could not perform formal meta-regression or age-stratified subgroup analyses to separate the potential confounding effect of age from the treatment effect. This imbalance therefore represents an important source of potential bias, and the between-group differences should be interpreted with this limitation in mind.

The reliability and generalizability of the findings may also be influenced by publication bias and clinical heterogeneity. Some endpoints occurred very infrequently, and several studies reported zero events. In addition, the small number of included studies and the predominance of non-randomized comparisons made it difficult to fully assess the stability of the pooled estimates. These factors may have affected the precision of the results, particularly for safety endpoints.

The included studies also varied substantially in sample size. The study by Dello Russo et al. (13) contributed a disproportionately large weight to the pooled estimates because it included a much larger cohort than the other studies. Although the leave-one-out sensitivity analyses showed that the direction and statistical significance of the main findings remained consistent, the influence of a single large study should still be acknowledged. Additional large-scale multicenter trials are needed to confirm whether the overall effect sizes observed in this analysis remain stable across broader clinical settings.

This study did not further stratify the analysis according to specific vHPSD or PFA ablation protocols, which is a clear limitation. The vHPSD group included protocols with different power and duration settings, such as QDOT Micro at 90 W for 4 s and FlexAbility at 70 W for 7 s. The PFA group also included different catheter designs, including flower-shaped and focal ablation catheters. Pooling these approaches helped preserve statistical power, but it also limited the ability to interpret device- or protocol-specific effects. We did not conduct subgroup analyses based only on a single parameter such as ablation power because power settings do not fully reflect how ablation is performed in real procedures. Operators often shorten the ablation duration or reduce the output on the posterior wall near the esophagus, while anterior wall ablation may be closer to the nominal protocol settings. A simple subgroup division based on power alone could therefore create groups that appear distinct on paper but remain affected by substantial residual confounding in practice.

Overall, the currently available evidence suggests that PFA and vHPSD may be broadly comparable in terms of efficacy and safety. However, this conclusion should be viewed as preliminary because the available evidence remains limited by study design, sample size, monitoring strategy, baseline imbalance, endpoint rarity, and heterogeneity in ablation protocols. Future studies should prioritize large-sample randomized controlled trials with longer follow-up periods. These studies should also provide stratified results according to AF type, patient characteristics, and specific ablation protocols so that clinicians can better judge which patients are most likely to benefit from each strategy.

## 6 Clinical implications and future prospects

The results of this study suggest that the comparison between PFA and vHPSD should not be limited to the single index of “postoperative recurrence or not”, but should include a more comprehensive and multi-dimensional end point. Future studies can further improve the evidence-based medicine in this field from the following dimensions: First, multi-center, large-sample randomized controlled trials should be conducted to directly compare the efficacy and safety of PFA and vHPSD, and prespecified subgroups should be stratified according to the type of paroxysmal/persistent AF to clarify the technical differences in different populations. Second, the definition and follow-up strategy of AF recurrence should be unified, and the application of long-term continuous ECG monitoring or implantable ECG monitoring devices should be promoted to reduce the missed detection of asymptomatic AF recurrence and improve the accuracy of recurrence assessment. Third, in addition to the success rate of acute pulmonary vein isolation (PVI), more core electrophysiological endpoints were reported, including the rate of primary PVI, the rate of pulmonary vein electrical connection at reablation, the durability of linear ablation lesions, and the long-term stability of left atrial posterior wall isolation. Fourth, a more comprehensive safety evaluation framework should be established to actively monitor the potential PAFA-specific adverse reactions, including PAF-related hemolysis, hemoglobinuria, renal injury, and coronary artery spasm, in addition to routine ablation complications, and to improve the long-term safety evidence of PFA. Fifth, the confounding variables such as learning curve, three-dimensional mapping application, intracardic ultrasound guidance, low fluoroscopy procedure and central operation experience should be reported to more accurately distinguish the inherent differences between the two technologies and the differences caused by the operation procedure and the operator's experience. Sixth, for patients with persistent AF and postoperative recurrent AF, the long-term efficacy and safety of PFA and vHPSD in left atrial posterior wall isolation, left atrial linear ablation, complex matrix modification and repeat ablation strategies were compared to fill the evidence gap in complex population.

## 7 Conclusion

Combined with the existing research evidence, this study showed that there was no significant difference in the efficacy and safety of catheter ablation for atrial fibrillation between pulsed field ablation (PFA) and ultra-high power short-time ablation (vHPSD). In the index of single complication (pericarditis), the PFA group was significantly lower than the vHPSD group. In addition, the fluoroscopy time and dose of the vHPSD group were higher than those of the VHPSD group. In view of the small sample size and short follow-up time, more randomized controlled trials with large sample size and long-term follow-up are still needed to further verify the above conclusions.

## Data Availability

The original contributions presented in the study are included in the article/Supplementary Material, further inquiries can be directed to the corresponding author/s.
