# Supplementary material for: Efficacy and safety of pulsed field ablation and very high-power short-duration ablation for atrial fibrillation: a meta-analysis
Source: Front Cardiovasc Med. 2026 Jul 21;13:1860269. doi: 10.3389/fcvm.2026.1860269 (PMC13433309; doi:10.3389/fcvm.2026.1860269)
Supplement: Supplementary file 1 [file Supplementaryfile1.docx]

**Supplementary Materials**

**Supplementary Table 1** Search strategy.

Embase

('atrial fibrillation'/exp OR 'atrium fibrillation':ti,ab,kw OR 'auricular fibrillation':ti,ab,kw OR 'cardiac atrial fibrillation':ti,ab,kw OR 'cardiac atrium fibrillation':ti,ab,kw OR 'fibrillation, heart atrium':ti,ab,kw OR 'heart atrial fibrillation':ti,ab,kw OR 'heart atrium fibrillation':ti,ab,kw OR 'heart fibrillation atrium':ti,ab,kw OR 'non-valvular atrial fibrillation':ti,ab,kw OR 'nonvalvular atrial fibrillation':ti,ab,kw OR 'atrial fibrillation':ti,ab,kw OR 'Atrial Fibrillations':ti,ab,kw OR 'Fibrillation, Atrial':ti,ab,kw OR 'Fibrillations, Atrial':ti,ab,kw OR 'Auricular Fibrillation':ti,ab,kw OR 'Auricular Fibrillations':ti,ab,kw OR 'Fibrillation, Auricular':ti,ab,kw OR 'Fibrillations, Auricular':ti,ab,kw OR 'Persistent Atrial Fibrillation':ti,ab,kw OR 'Atrial Fibrillation, Persistent':ti,ab,kw OR 'Atrial Fibrillations, Persistent':ti,ab,kw OR 'Fibrillation, Persistent Atrial':ti,ab,kw OR 'Fibrillations, Persistent Atrial':ti,ab,kw OR 'Persistent Atrial Fibrillations':ti,ab,kw OR 'Familial Atrial Fibrillation':ti,ab,kw OR 'Atrial Fibrillation, Familial':ti,ab,kw OR 'Atrial Fibrillations, Familial':ti,ab,kw OR 'Familial Atrial Fibrillations':ti,ab,kw OR 'Fibrillation, Familial Atrial':ti,ab,kw OR 'Fibrillations, Familial Atrial':ti,ab,kw OR 'Paroxysmal Atrial Fibrillation':ti,ab,kw OR 'Atrial Fibrillation, Paroxysmal':ti,ab,kw OR 'Atrial Fibrillations, Paroxysmal':ti,ab,kw OR 'Fibrillation, Paroxysmal Atrial':ti,ab,kw OR 'Fibrillations, Paroxysmal Atrial':ti,ab,kw OR 'Paroxysmal Atrial Fibrillations') AND ('pulsed field ablation'/exp OR 'pulsed field ablation catheter'/exp OR 'pulsed field ablation system'/exp OR 'pulsed field ablation therapy'/exp OR 'pulsed-field ablation':ti,ab,kw OR 'pulse field ablation':ti,ab,kw OR 'PF ablation':ti,ab,kw OR 'pulsed electric field ablation':ti,ab,kw OR 'PEF ablation':ti,ab,kw OR 'electroporation ablation':ti,ab,kw OR 'pulsed field ablation catheter':ti,ab,kw OR 'pulsed field ablation system':ti,ab,kw OR 'pulsed field ablation therapy':ti,ab,kw OR 'PFA':ti,ab,kw OR 'electroporation':ti,ab,kw OR 'no-thermal ablation':ti,ab,kw OR 'irreversible electroporation':ti,ab,kw OR 'IRE':ti,ab,kw) AND ('radiofrequency ablation':ti,ab,kw OR 'RF ablation':ti,ab,kw OR 'RFA':ti,ab,kw OR 'RFA therapy':ti,ab,kw OR 'Radiofrequency Ablation':ti,ab,kw OR 'Ablation, Radiofrequency':ti,ab,kw OR 'Radio-Frequency Ablation':ti,ab,kw OR 'Ablation, Radio-Frequency':ti,ab,kw OR 'Radio Frequency Ablation':ti,ab,kw OR 'Ablation, Radio Frequency':ti,ab,kw OR 'very high power short duration':ti,ab,kw OR 'very high-power short-duration ablation':ti,ab,kw OR 'vHPSD':ti,ab,kw OR 'VHPSD':ti,ab,kw OR 'QDOT':ti,ab,kw OR 'QMODE+':ti,ab,kw OR 'thermal ablation':ti,ab,kw OR 'QDOT Micro':ti,ab,kw OR '90 W/4 s':ti,ab,kw OR 'vHP-SD':ti,ab,kw OR 'vHPvSD':ti,ab,kw OR 'temperature-controlled ablation':ti,ab,kw )

Pubmed

((("Atrial Fibrillation"[Mesh]) OR ((Atrial Fibrillations[Title/Abstract]) OR (Fibrillation, Atrial[Title/Abstract]) OR (Fibrillations, Atrial[Title/Abstract]) OR (Auricular Fibrillation[Title/Abstract]) OR (Auricular Fibrillations[Title/Abstract]) OR (Fibrillation, Auricular[Title/Abstract]) OR (Fibrillations, Auricular[Title/Abstract]) OR (Persistent Atrial Fibrillation[Title/Abstract]) OR (Atrial Fibrillation, Persistent[Title/Abstract]) OR (Atrial Fibrillations, Persistent[Title/Abstract]) OR (Fibrillation, Persistent Atrial[Title/Abstract]) OR (Fibrillations, Persistent Atrial[Title/Abstract]) OR (Persistent Atrial Fibrillations[Title/Abstract]) OR (Familial Atrial Fibrillation[Title/Abstract]) OR (Atrial Fibrillation, Familial[Title/Abstract]) OR (Atrial Fibrillations, Familial[Title/Abstract]) OR (Familial Atrial Fibrillations[Title/Abstract]) OR (Fibrillation, Familial Atrial[Title/Abstract]) OR (Fibrillations, Familial Atrial[Title/Abstract]) OR (Paroxysmal Atrial Fibrillation[Title/Abstract]) OR (Atrial Fibrillation, Paroxysmal[Title/Abstract]) OR (Atrial Fibrillations, Paroxysmal[Title/Abstract]) OR (Fibrillation, Paroxysmal Atrial[Title/Abstract]) OR (Fibrillations, Paroxysmal Atrial[Title/Abstract]) OR (Paroxysmal Atrial Fibrillations[Title/Abstract])(atrium fibrillation[Title/Abstract]) OR (auricular fibrillation[Title/Abstract]) OR (cardiac atrial fibrillation[Title/Abstract]) OR (cardiac atrium fibrillation[Title/Abstract]) OR (fibrillation, heart atrium[Title/Abstract]) OR (heart atrial fibrillation[Title/Abstract]) OR (heart atrium fibrillation[Title/Abstract]) OR (heart fibrillation atrium[Title/Abstract]) OR (non-valvular atrial fibrillation[Title/Abstract]) OR (nonvalvular atrial fibrillation[Title/Abstract]))) AND ((pulsed field ablation[Title/Abstract]) OR (pfa[Title/Abstract]) OR (PFA[Title/Abstract]) OR (Pulsed Field Ablation[Title/Abstract]) OR (Pulsed Electric Field[Title/Abstract]) OR (pulsed field ablation catheter[Title/Abstract]) OR (pulsed field ablation system[Title/Abstract]) OR (pulsed field ablation therapy[Title/Abstract]) OR (pulsed-field ablation[Title/Abstract]) OR (pulse field ablation[Title/Abstract]) OR (PF ablation[Title/Abstract]) OR (pulsed electric field ablation[Title/Abstract]) OR (PEF ablation[Title/Abstract]) OR (electroporation ablation[Title/Abstract]) OR (pulsed field ablation catheter[Title/Abstract]) OR (pulsed field ablation system[Title/Abstract]) OR (pulsed field ablation therapy[Title/Abstract]) OR (electroporation[Title/Abstract]) OR (irreversible electroporation[Title/Abstract]) OR (IRE[Title/Abstract])) AND (("Radiofrequency Ablation"[Mesh]) OR ((Ablation, Radiofrequency[Title/Abstract]) OR (Radio-Frequency Ablation[Title/Abstract]) OR (Ablation, Radio-Frequency[Title/Abstract]) OR (Radio Frequency Ablation[Title/Abstract]) OR (Ablation, Radio Frequency[Title/Abstract]) OR (very high power short duration[Title/Abstract]) OR (very high-power short-duration ablation[Title/Abstract]) OR (vHPSD[Title/Abstract]) OR (VHPSD[Title/Abstract]) OR (QDOT[Title/Abstract]) OR (QMODE+[Title/Abstract]) OR (thermal ablation[Title/Abstract]) OR (QDOT Micro[Title/Abstract]) OR (90 W/4 s[Title/Abstract]) OR (vHP-SD[Title/Abstract]) OR (vHPvSD[Title/Abstract]) OR (temperature-controlled ablation[Title/Abstract])))

Cochrane library

((MeSH descriptor: [Atrial Fibrillation] explode all trees) OR ((Atrial Fibrillations, Familial):ti,ab,kw OR (Familial Atrial Fibrillations):ti,ab,kw OR (Familial Atrial Fibrillation):ti,ab,kw OR (Atrial Fibrillation, Familial):ti,ab,kw OR (Fibrillation, Familial Atrial):ti,ab,kw OR (Fibrillations, Familial Atrial):ti,ab,kw OR (Fibrillations, Persistent Atrial):ti,ab,kw OR (Atrial Fibrillations, Persistent):ti,ab,kw OR (Fibrillation, Persistent Atrial):ti,ab,kw OR (Persistent Atrial Fibrillation):ti,ab,kw OR (Persistent Atrial Fibrillations):ti,ab,kw OR (Atrial Fibrillation, Persistent):ti,ab,kw OR (Paroxysmal Atrial Fibrillation):ti,ab,kw OR (Fibrillation, Paroxysmal Atrial):ti,ab,kw OR (Atrial Fibrillation, Paroxysmal):ti,ab,kw OR (Fibrillations, Paroxysmal Atrial):ti,ab,kw OR (Atrial Fibrillations, Paroxysmal):ti,ab,kw OR (Paroxysmal Atrial Fibrillations):ti,ab,kw OR (Atrial Fibrillations):ti,ab,kw OR (Auricular Fibrillation):ti,ab,kw OR (Auricular Fibrillations):ti,ab,kw OR (Fibrillation, Auricular):ti,ab,kw OR (Fibrillation, Atrial):ti,ab,kw OR (Fibrillations, Auricular):ti,ab,kw OR (Fibrillations, Atrial):ti,ab,kw)) AND ((MeSH descriptor: [Catheter Ablation] explode all trees) OR ((Ablation, Catheter):ti,ab,kw OR (Ablation, Electrical Catheter):ti,ab,kw OR (Catheter Ablation, Electric):ti,ab,kw OR (Electrical Catheter Ablation):ti,ab,kw OR (Ablation, Electric Catheter):ti,ab,kw OR (Electric Catheter Ablation):ti,ab,kw OR (Catheter Ablation, Electrical):ti,ab,kw OR (Ablation, Percutaneous Catheter):ti,ab,kw OR (Percutaneous Catheter Ablation):ti,ab,kw OR (Catheter Ablation, Percutaneous):ti,ab,kw OR (Transvenous Electric Ablation):ti,ab,kw OR (Electrical Ablation, Transvenous):ti,ab,kw OR (Transvenous Electrical Ablation):ti,ab,kw OR (Electric Ablation, Transvenous):ti,ab,kw OR (Ablation, Transvenous Electric):ti,ab,kw OR (Ablation, Transvenous Electrical):ti,ab,kw OR (Transvenous Catheter Ablation):ti,ab,kw OR (Ablation, Transvenous Catheter):ti,ab,kw OR (Catheter Ablation, Transvenous):ti,ab,kw OR (Radiofrequency Catheter Ablation):ti,ab,kw OR (Ablation, Radiofrequency Catheter):ti,ab,kw OR (Catheter Ablation, Radiofrequency) OR (very high power short duration):ti,ab,kw OR (very high-power short-duration ablation):ti,ab,kw OR (vHPSD):ti,ab,kw OR (VHPSD):ti,ab,kw OR (QDOT):ti,ab,kw OR (QMODE):ti,ab,kw OR (thermal ablation):ti,ab,kw OR (QDOT Micro):ti,ab,kw OR (90 W 4 s):ti,ab,kw OR (vHPSD):ti,ab,kw OR (vHPvSD):ti,ab,kw OR (temperature controlled ablation):ti,ab,kw)) AND ((pulsed field ablation):ti,ab,kw OR (pfa):ti,ab,kw OR (PFA):ti,ab,kw OR (Pulsed Field Ablation):ti,ab,kw OR (Pulsed Electric Field):ti,ab,kw OR (pulsed field ablation catheter):ti,ab,kw OR (pulsed field ablation system):ti,ab,kw OR (pulsed field ablation therapy):ti,ab,kw OR (pulsed-field ablation):ti,ab,kw OR (pulse field ablation):ti,ab,kw OR (PF ablation):ti,ab,kw OR (pulsed electric field ablation):ti,ab,kw OR (PEF ablation):ti,ab,kw OR (electroporation ablation):ti,ab,kw OR (pulsed field ablation catheter):ti,ab,kw OR (pulsed field ablation system):ti,ab,kw OR (pulsed field ablation therapy):ti,ab,kw OR (electroporation):ti,ab,kw OR (irreversible electroporation):ti,ab,kw OR (IRE):ti,ab,kw)

Web of science

''Atrial Fibrillation'' OR ''Atrial Fibrillations'' OR ''Fibrillation, Atrial'' OR ''Fibrillations, Atrial'' OR ''Auricular Fibrillation'' OR ''Auricular Fibrillations'' OR ''Fibrillation, Auricular'' OR ''Fibrillations, Auricular'' OR ''Persistent Atrial Fibrillation'' OR ''Atrial Fibrillation, Persistent'' OR ''Atrial Fibrillations, Persistent'' OR ''Fibrillation, Persistent Atrial'' OR ''Fibrillations, Persistent Atrial'' OR ''Persistent Atrial Fibrillations'' OR ''Familial Atrial Fibrillation'' OR ''Atrial Fibrillation, Familial'' OR ''Atrial Fibrillations, Familial'' OR ''Familial Atrial Fibrillations'' OR ''Fibrillation, Familial Atrial'' OR ''Fibrillations, Familial Atrial'' OR ''Paroxysmal Atrial Fibrillation'' OR ''Atrial Fibrillation, Paroxysmal'' OR ''Atrial Fibrillations, Paroxysmal'' OR ''Fibrillation, Paroxysmal Atrial'' OR ''Fibrillations, Paroxysmal Atrial'' OR ''Paroxysmal Atrial Fibrillations'' (Topic) and ''pulsed field ablation'' OR '''pulsed-field ablation'' OR ''pulse field ablation'' OR ''PF ablation'' OR ''pulsed electric field ablation'' OR ''PEF ablation'' OR ''electroporation ablation'' OR ''pulsed field ablation catheter'' OR ''pulsed field ablation system'' OR ''pulsed field ablation therapy'' OR ''PFA'' OR ''electroporation'' OR ''no-thermal ablation'' OR ''irreversible electroporation'' OR ''IRE'' (Topic) and ''Radiofrequency Ablation'' OR ''Ablation, Radiofrequency'' OR ''Radio-Frequency Ablation'' OR ''Ablation, Radio-Frequency'' OR ''Radio Frequency Ablation'' OR ''Ablation, Radio Frequency'' OR ''very high power short duration'' OR ''very high-power short-duration ablation'' OR ''vHPSD'' OR ''VHPSD'' OR ''QDOT'' OR ''QMODE+'' OR ''thermal ablation'' OR ''QDOT Micro'' OR ''90 W/4 s'' OR ''vHP-SD'' OR ''vHPvSD'' OR ''temperature-controlled ablation'' (Topic)

万方

主题:("心房颤动" OR "房颤" OR "心房纤颤" OR "AF" OR "房纤" OR "心房颤动病" OR "心房纤维性颤动" OR "心房纤维颤动" OR "心房性颤动" OR "阵发性心房颤动" OR "阵发性房颤" OR "阵发性心房纤颤" OR "持续性心房颤动" OR "持续性房颤" OR "持续性心房纤颤" OR "非阵发性心房颤动" OR "非阵发性房颤" OR "非阵发性心房纤颤") and 主题:("脉冲电场消融" OR "脉冲场消融" OR "PFA" OR "不可逆电穿孔" OR "IRE" OR "电穿孔消融" OR "脉冲电消融" OR "电场消融" OR "双相脉冲电场消融" OR "双相脉冲场消融" OR "双极脉冲电场消融" OR "单相脉冲电场消融" OR "单相脉冲场消融" OR "微秒脉冲电场消融" OR "高频不可逆电穿孔" OR "法拉脉冲" OR "Farapulse" OR "PulseSelect" ) and 主题:("超高功率短时程消融" OR "超高功率短时消融" OR "vHPSD" OR "VHPSD" OR "vHP-SD" OR "极高功率短时程消融" OR "极高功率短时消融" OR "极高压短时消融" OR "超高速射频消融" OR "短时程超高功率消融" OR "超高功率射频消融" OR "90 瓦 4 秒消融" OR "90W/4s 消融" OR "90 瓦 4 秒射频消融" OR "QDOT 消融" OR "QDOT 导管" OR "QDOT MICRO 导管" OR "QMODE+" OR "QMODE 模式" OR "智能温控超高功率消融导管")

维普

(U="心房颤动" OR "房颤" OR "心房纤颤" OR "AF" OR "房纤" OR "心房颤动病" OR "心房纤维性颤动" OR "心房纤维颤动" OR "心房性颤动" OR "阵发性心房颤动" OR "阵发性房颤" OR "阵发性心房纤颤" OR "持续性心房颤动" OR "持续性房颤" OR "持续性心房纤颤" OR "非阵发性心房颤动" OR "非阵发性房颤" OR "非阵发性心房纤颤") AND (U="脉冲电场消融" OR "脉冲场消融" OR "PFA" OR "不可逆电穿孔" OR "IRE" OR "电穿孔消融" OR "脉冲电消融" OR "电场消融" OR "双相脉冲电场消融" OR "双相脉冲场消融" OR "双极脉冲电场消融" OR "单相脉冲电场消融" OR "单相脉冲场消融" OR "微秒脉冲电场消融" OR "高频不可逆电穿孔" OR "法拉脉冲" OR "Farapulse" OR "PulseSelect") AND (U="超高功率短时程消融" OR "超高功率短时消融" OR "vHPSD" OR "VHPSD" OR "vHP-SD" OR "极高功率短时程消融" OR "极高功率短时消融" OR "极高压短时消融" OR "超高速射频消融" OR "短时程超高功率消融" OR "超高功率射频消融" OR "90 瓦 4 秒消融" OR "90W/4s 消融" OR "90 瓦 4 秒射频消融" OR "QDOT 消融" OR "QDOT 导管" OR "QDOT MICRO 导管" OR "QMODE+" OR "QMODE 模式" OR "智能温控超高功率消融导管")

知网

SU=("心房颤动" + "房颤" + "心房纤颤" + "AF" + "房纤" + "心房颤动病" + "心房纤维性颤动" + "心房纤维颤动" + "心房性颤动" + "阵发性心房颤动" + "阵发性房颤" + "阵发性心房纤颤" + "持续性心房颤动" + "持续性房颤" + "持续性心房纤颤" + "非阵发性心房颤动" + "非阵发性房颤) AND SU=("脉冲电场消融" + "脉冲场消融" + "PFA" + "不可逆电穿孔" + "IRE" + "电穿孔消融" + "脉冲电消融" + "电场消融" + "双相脉冲电场消融" + "双相脉冲场消融" + "双极脉冲电场消融" + "单相脉冲电场消融" + "单相脉冲场消融" + "微秒脉冲电场消融" + "高频不可逆电穿孔" + "法拉脉冲" + "Farapulse" + "PulseSelect" ) AND SU=("超高功率短时程消融" + "超高功率短时消融" + "vHPSD" + "VHPSD" + "vHP-SD" + "极高功率短时程消融" + "极高功率短时消融" + "极高压短时消融" + "超高速射频消融" + "短时程超高功率消融" + "超高功率射频消融" + "90 瓦 4 秒消融" + "90W/4s 消融" + "90 瓦 4 秒射频消融" + "QDOT 消融" + "QDOT 导管" + "QDOT MICRO 导管" + "QMODE+" + "QMODE 模式" + "智能温控超高功率消融导管")

**Supplementary Table 2** Summary of Conclusions Following Sensitivity Analysis with Alternative Models

| **Outcome** | **Main analysis** | **Fixed effects model (Mantel-Haenszel, MH/ Inverse Variance)** | **Random effects model (DerSimonian-Laird, DL)** | **Random effects model (Paule-Mandel, PM)** | **Interpretation** |
| --- | --- | --- | --- | --- | --- |
| Freedom from arrhythmia | RR =0.99, 95%CI: 0.91 to 1.07 | RR =0.981, 95%CI: 0.929 to 1.035 | RR =0.988, 95%CI: 0.919 to 1.062 | RR =0.984, 95%CI: 0.920-1.052 | Robust |
| Immediate pulmonary vein isolation rate | RR =1.00, 95%CI: 0.90 to 1.10 | RR =1.0003, 95%CI: 0.9910 to 1.0096 | RR =1.0000, 95%CI: 0.9922 to 1.0079 | RR =1.0000, 95%CI: 0.9922 to 1.0079 | Robust |
| Total incidence of surgery-related complications | RR =0.89, 95%CI: 0.58 to 1.35 | RR =0.85, 95%CI: 0.51 to 1.43 | RR =0.89, 95%CI: 0.52 to 1.51 | RR =0.89, 95%CI: 0.52 to 1.51 | Robust |
| Cardiac tamponade | RR =1.19, 95%CI: 0.29 to 4.88 | RR =1.221, 95%CI: 0.304 to 4.908 | RR =1.190, 95%CI: 0.290 to 4.884 | RR =1.190, 95%CI: 0.290 to 4.884 | Robust |
| Stroke | RR =0.47, 95%CI: 0.08 to 2.66 | RR =0.453, 95%CI: 0.087 to 2.373 | RR =0.469, 95%CI: 0.083 to 2.657 | RR =0.469, 95%CI: 0.083 to 2.657 | Robust |
| Vascular complications | RR =1.35, 95%CI: 0.56 to 3.25 | RR =1.284, 95%CI: 0.610to2.703 | RR =1.386, 95%CI: 0.444 to 4.331 | RR =1.393, 95%CI:0.398 to 4.869 | Robust |
| Pericarditis | RR =0.15, 95%CI: 0.03 to 0.84 | RR =0.140, 95%CI: 0.025 to 0.776 | RR =0.151, 95%CI: 0.027 to 0.836 | RR =0.151, 95%CI: 0.027 to 0.836 | Robust |
| Procedure time | MD=-31.64,95%CI: -40.22 to -23.06 | MD =-27.78, 95%CI: -30.23 to -25.32 | MD =-31.52, 95%CI: -38.16 to -24.88 | MD =-31.70, 95%CI: -39.18 to -24.23 | Robust |
| Fluoroscopy time | MD=6.93,95%CI: 4.32 to 9.55 | MD =-5.82, 95%CI: 5.28 to 6.36 | MD =-6.88 95%CI: 4.91 to 8.86 | MD =0.140, 95%CI: -39.18 to -24.23 | Robust |

**Supplementary Table 3** Summary of Publication Bias Test Results for Individual Complications

| complication | n | Egger test | | Begg test | | Peters test | |
| --- | --- | --- | --- | --- | --- | --- | --- |
|  |  | Z | P | Z | P | Z | P |
| Cardiac tamponade | 3 | 1.133 | 0.4603 | 1.000 | 0.3333 | -0.566 | 0.6722 |
| stroke | 3 | -1.540 | 0.3666 | -0.333 | 1.000 | -0.125 | 0.9210 |
| Vascular complications | 5 | 0.101 | 0.9262 | 0.200 | 0.8167 | 0.700 | 0.5346 |
| Pericarditis | 2 | NA | NA | NA | NA | NA | NA |

A

B

C

D

E

F

G

H

I

J

**Supplementary Figure 1** Forest plots comparing baseline characteristics between the PFA and vHPSD groups (Primary Analysis)

(A) Age; (B) Sex; (C) BMI; (D) Types of atrial fibrillation; (E) Hypertension; (F) Diabetes; (G) Coronary heart disease; (H) LVEF; (I) Left Atrial Size; (J) CHA2DS2-VASc Score.

A

B

C

D

E

F

G

**Supplementary Figure 2** Forest plot of baseline characteristics between the PFA and vHPSD groups (Sensitivity Analysis)

Forest plots display the pooled estimates after excluding studies identified as potential sources of heterogeneity.

(A) Sex; (B) Types of atrial fibrillation; (C) Diabetes; (D) Coronary Heart Disease; (E) LVEF; (F) Left Atrial Size; (G) CHA2DS2-VASc Score.

| Freedom from arrhythmia |  |
| --- | --- |
| Immediate pulmonary vein isolation rate |  |
| Total incidence of surgery-related complications |  |
| Procedure time |  |
| Fluoroscopy time |  |
|  |  |

**Supplementary Figure 3** Forest Plot Summary Excluding Conference Abstracts

| Freedom from arrhythmia |  |
| --- | --- |
| Immediate pulmonary vein isolation rate |  |
| Total incidence of surgery-related complications |  |
| Incidence of single complication |  |
| Procedure time |  |
| Fluoroscopy time |  |

**Supplementary Figure 4** Forest Plot Summary After Replacing the Effect Size Measure

| Freedom from arrhythmia |  |
| --- | --- |
| Immediate pulmonary vein isolation rate |  |
| Total incidence of surgery-related complications |  |
| Incidence of single complication |  |
| Procedure time |  |
| Fluoroscopy time |  |

**Supplementary Figure 5** Summary Plot of the Leave-One-Out Analysis
